# A Comprehensive In Silico Method to Study the QSTR of the Aconitine Alkaloids for Designing Novel Drugs

**DOI:** 10.3390/molecules23092385

**Published:** 2018-09-18

**Authors:** Ming-Yang Wang, Jing-Wei Liang, Kamara Mohamed Olounfeh, Qi Sun, Nan Zhao, Fan-Hao Meng

**Affiliations:** School of Pharmacy, China Medical University, Liaoning 110122, China; wmy940623@163.com (M.-Y.W.); jwliang@cmu.edu.cn (J.-W.L.); mohamedkamara6994@yahoo.com (K.M.O.); Jilindaxuesunqi@126.com (Q.S.); zhaonan-dd@163.com (N.Z.)

**Keywords:** aconitine, quantitative structure–toxicity relationship (QSTR), docking, network, alkaloids

## Abstract

A combined in silico method was developed to predict potential protein targets that are involved in cardiotoxicity induced by aconitine alkaloids and to study the quantitative structure–toxicity relationship (QSTR) of these compounds. For the prediction research, a Protein-Protein Interaction (PPI) network was built from the extraction of useful information about protein interactions connected with aconitine cardiotoxicity, based on nearly a decade of literature and the STRING database. The software Cytoscape and the PharmMapper server were utilized to screen for essential proteins in the constructed network. The Calcium-Calmodulin-Dependent Protein Kinase II alpha (CAMK2A) and gamma (CAMK2G) were identified as potential targets. To obtain a deeper insight on the relationship between the toxicity and the structure of aconitine alkaloids, the present study utilized QSAR models built in Sybyl software that possess internal robustness and external high predictions. The molecular dynamics simulation carried out here have demonstrated that aconitine alkaloids possess binding stability for the receptor CAMK2G. In conclusion, this comprehensive method will serve as a tool for following a structural modification of the aconitine alkaloids and lead to a better insight into the cardiotoxicity induced by the compounds that have similar structures to its derivatives.

## 1. Introduction

The rhizomes and roots of aconitine species, a genus of the family Ranunculaceae, are commonly used in treatment for various illnesses such as collapse, syncope, rheumatic fever, joints pain, gastroenteritis, diarrhea, edema, bronchial asthma, and tumors. They are also involved in the management of endocrinal disorders such as irregular menstruation [1,2]. However, the usefulness of this aconitine species component intermingles with toxicity after it is administered to a diseased patient. So far, few articles have recorded the misuse of aconitine medicinals with strong emphasis and thus have referenced that the misuse of this medicinal can result in severe cardio- and neurotoxicity [3,4,5,6,7]. In our past research, it was evidenced that the aconitine component is the main active ingredient in this species’ root and rhizome, and is responsible for both therapeutic and toxic effects [8].

This medicinal has been tested for cancerological and dermatological activities. Its application to disease conditions proved to exhibit an activity that slowed down cancer tumor growth and to cure serious cases of dermatosis. It was also found to have an effect on postoperative analgesia [9,10,11,12]. However, a previous safety study has revealed that aconitine toxicity is responsible for its restriction in clinical settings. Further studies are needed to explain the cause of aconitine toxicity as well as to show whether the toxicity supersedes its usefulness. A combined network analysis and in silico study was once performed to obtain insight on the relationship between aconitine alkaloid toxicity and the aconitine structure, and it was found that the cardiotoxicity of aconitine is the primary cause of patient death. The aconitine poison is similar to the poison created by some pivotal proteins such as the ryanodine receptor (RYR1 and RYR2), the Gap junction α-1 protein (GJA1), and the sodium–calcium exchanger (SLC8A1) [9,10,11,12]. However, among all existing studies about the aconitine medicinal, no one has reported detail of its specific binding target protein linked to toxicity.

Protein–Protein Interactions (PPIs) participate in many metabolic processes occurring in living organisms such as the cellular communication, immunological response, and gene expression control [13,14]. A systematic description of these interactions aids in the elucidation of interrelationships among targets. The targeting of PPIs with small-molecule compounds is becoming an essential step in a mechanism study [14]. The present study was designed and undertaken to identify the critical protein that can affect the cardiotoxicity of aconitine alkaloids. A PPI network built by the STRING database is a physiological contact for the high specificity that has been established for several protein molecules and has stemmed from computational prediction, knowledge transfer between organisms, and interactions aggregated from other databases [15]. The analysis of the PPI network is based on nodes and edges and is always performed via cluster analysis and centrality measurements [16,17]. In cluster analysis, highly interconnected nodes and protein target nodes are divided and used to form sub-graphs. The reliability of the PPI network is identified by the content of each sub-graph [18]. The variability in centrality measurements is connected to the quantitative relationship between the protein targets and its weightiness in the network [18]. Hence, PPI networks with protein targets related to aconitine alkaloid cardiotoxicity must enable us to find the most relevant protein for aconitine toxicity and to understand the mechanism at the network level. In our research, the evaluation and visualization analysis of essential proteins related to cardiotoxicity in PPIs were performed by the ClusterONE and CytoNCA plugins in Cytoscape 3.5, designed to find the potential protein targets via combination with conventional integrated pharmacophore matching technology built in the PharmMapper platform.

Structural modification of a familiar natural product, active compound, or clinical drug is an efficient method for designing a novel drug. The main purpose of the structural modification is to reduce the toxicity of the target compound while enhancing the utility of the drug [19]. The identification of the structure–function relationship is an essential step in the drug discovery and design, the determination of the 3D protein structures was the key step in identifying the internal interactions in the ligand–receptor complexes. X-ray crystallography and NMR were the only accepted techniques of determining the 3D protein structure. Although the 3D structure obtained by these two powerful techniques are accurate and reliable, they are time-consuming and costly [20,21,22,23,24]. With the rapid development of structural bioinformatics and Computer-Aided Drug Design (CADD) techniques in the last decade, computational structures are becoming increasingly reliable. The application of structural bioinformatics and CADD techniques can improve the efficiency of this process [25,26,27,28,29,30,31,32,33,34]. The ligand-based quantitative structure–toxicity relationship (QSTR) and receptor-based docking technology are regarded as effective and useful tools in analysis of structure–function relationships [35,36,37,38]. The contour maps around aconitine alkaloids generated by comparative molecular field analysis (CoMFA) and comparative molecular similarity index analysis (CoMSIA) were combined with the interactions between ligand substituents and amino acids obtained from docking results to gain insight on the relationship between the structure of aconitine alkaloids and their toxicity.

Scores from functions were used to evaluate the docking result. The value-of-fit score in MOE software reflects the binding stability and affinity of the ligand–receptor complexes. When screening for the most potential target for cardiotoxicity, the experimental data was combined with the value-of-fit score by the NDCG (Normalized Discounted Cumulative Gain). The possibility of a protein being a target of cardiotoxicity corresponds with the consistency of this experimental data. 

**Figure 1:**
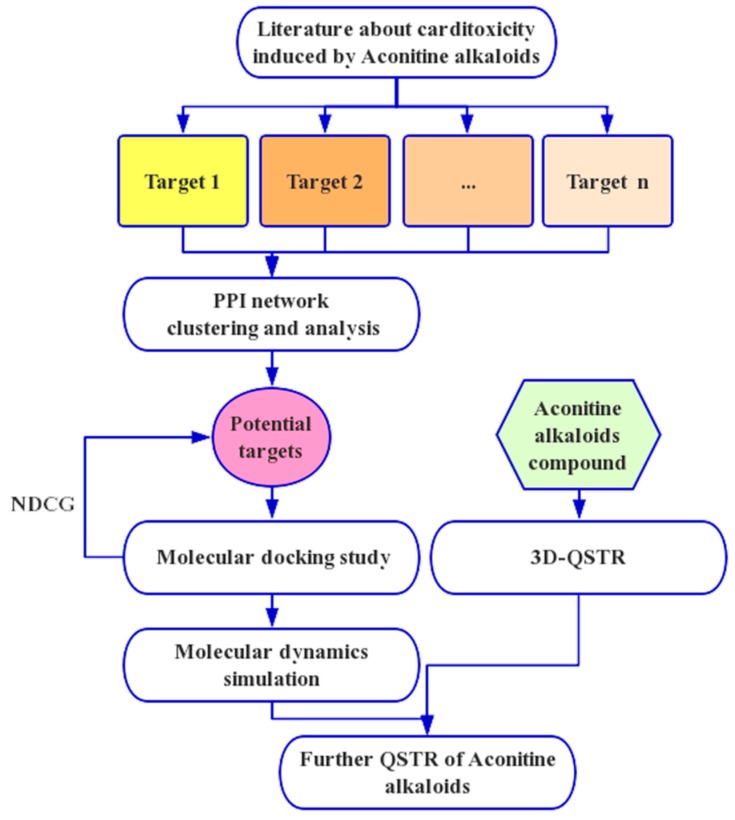
The whole framework of the comprehensive in silico method for screening potential targets and studying the quantitative structure–toxicity relationship (QSTR).

Since the pioneering paper entitled “The Biological Functions of Low-Frequency Phonons” [39] was published in 1977, many investigations of biomacromolecules from a dynamic point of view have occurred. These studies have suggested that low-frequency (or terahertz frequency) collective motions do exist in proteins and DNA [40,41,42,43,44]. Furthermore, many important biological functions in proteins and DNA and their dynamic mechanisms, such as cooperative effects [45], the intercalation of drugs into DNA [42], and the assembly of microtubules [46], have been revealed by studying the low-frequency internal motions, as summarized in a comprehensive review [40]. Some scientists have even applied this kind of low-frequency internal motion to medical treatments [47,48]. Investigation of the internal motion in biomacromolecules and its biological functions is deemed as a “genuinely new frontier in biological physics,” as announced in the mission of some biotech companies (see, e.g., Vermont Photonics). In order to consider the static structural information of the ligand–receptor complex, dynamical information should be also considered in the process of drug discovery [49,50]. Finally, molecular dynamics was carried out to verify the binding affinity and stability of aconitine alkaloids and the most potential target. This present study may be instrumental in our future studies for the synergism and attenuation of aconitine alkaloids and for the exploitation of its clinical application potential. A flowchart of procedures in our study is shown in Figure 1.

## 2. Results

The 33 compounds were aligned over, under the superimposition of the common moiety and Template Compound **6**. The statistical parameters for database alignment—q^2^, r^2^, F, and SEE—were calculated by Sybyl X2.0 as shown in Table 1. The CoMFA model with the optimal number of 6 components presented a q^2^ of 0.624, an r^2^ of 0.966, an F of 124.127, and an SEE of 0.043, and contributions of the steric and electrostatic fields were 0.621 and 0.379, respectively. The CoMSIA model with the optimal number of 4 components presented a q^2^ of 0.719, an r^2^ of 0.901, an F of 157.458, and an SEE of 0.116, and the contributions of steric, electrostatic, hydrophobic, hydrogen bond acceptor, and hydrogen bond donor fields were 0.120, 0.204, 0.327, 0.216, and 0.133, respectively. The statistical results proved that the aconitine alkaloids QSTR model of CoMFA and CoMSIA under the database alignment have adequate predictability.

**Figure 2:**
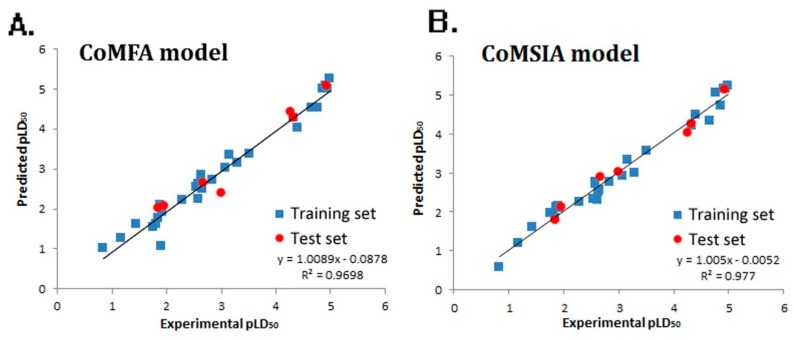
Experimental versus predicted activity of the training and test sets based on the comparative molecular field analysis (CoMFA) model (**A**) and comparative molecular similarity index analysis (CoMSIA) model (**B**).

Experimental and predicted pLD_50_ values of both the training set and test set are shown in Figure 2, and the CoMFA (Figure 2A) and CoMSIA (Figure 2B) model gave the correlation coefficient (r^2^) value of 0.9698 and 0.977, respectively, which demonstrated the internal robustness and external high prediction of the QSTR models.

**Figure 3:**
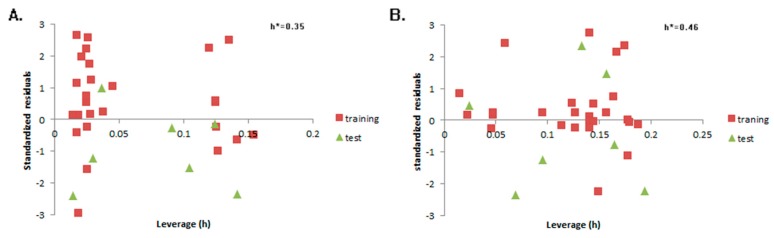
Residuals vs. Leverage Williams plots of the aconitine Topomer CoMFA (**A**) and CoMSIA (**B**) models.

Residuals vs. Leverage Williams plots of the aconitine QSTR models are shown in Figure 3A,B. All values of standardized residuals fall between 3σ and −3σ, and the values of leverage are less than h*, so the two models demonstrate potent extensibility and predictability.

**Figure 4:**
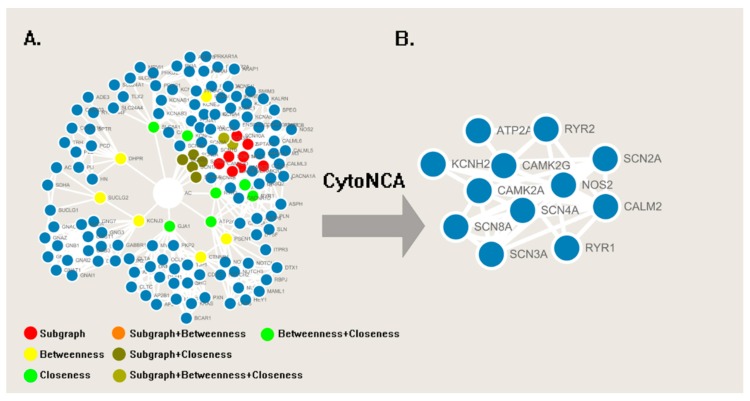
(**A**) The PPI network of proteins about cardiotoxicity induced by aconitine alkaloids. (**B**) The sub-network with essential protein generated from the Cytoscape and CytoNCA plugin.

Under MeSH (Medical Subject Headings), a total of 491 articles (261 articles were received from Web of Science, and others were received from PubMed) were retrieved. After selecting cardiotoxicity-related and excluding repetitive articles, 274 articles were used to extract the correlative proteins and pathways for building a PPI network in the STRING server. The correlative proteins or pathways are shown in Table 2. All proteins were taken as input protein in the STRING database to find its direct and functional partners [51], and proteins and its partners were then imported into the Cytoscape 3.5 to generate the PPI network with 148 nodes and 872 edges (Figure 4).

During the case of screening of the essential proteins in PPI network, three centrality measurements (Subgraph Centrality, Betweenness Centrality, and Closeness Centrality) in CytoNCA were utilized to evaluate the weight of nodes. After removing the central node “AC,” the centrality measurements of 147 nodes were calculated by CytoNCA and documented in Table S1. The top 10% of three centrality measurement values of all node are painted with a different color in Figure 4A. To screen the node with the high values of each three centrality measures, nodes with three colors were overlapped and merged into sub-networks in Figure 4B. 

**Figure 5:**
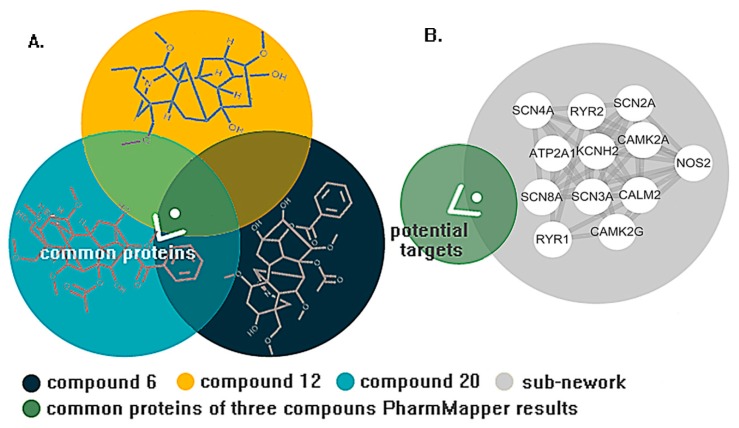
(**A**) Common targets of three aconitine alkaloids obtained from overlapping the PharmMapper results. (**B**) The potential target received from superimposing the PharmMapper and CytoNCA result.

In the sub-networks, the voltage-gated calcium and sodium channel accounted for a large proportion, which is consistent with our research in clustering the network (Clusters 1, 2, and 9). All proteins in the sub-networks will be utilized to predict the results of the PharmMapper server to receive the potential target of cardiotoxicity induced by aconitine alkaloids (in Figure 5A,B). In the meantime, 2V7O (CAMK2G) and 2VZ6 (CAMK2A) were identified as the potential targets with higher fit scores.

All compounds were docked into three potential targets. The values of nDCG are shown in Table 3. The Dock study of three proteins with an NDCG of 0.8503 and 0.9122, respectively (the detailed docking result is shown in Table S2) proves that the result of the Dock study of 2V7O is consistent with the Experimental pLD_50_, so the protein 2V7O was utilized for the ligand interaction analysis.

## 3. Discussion

The 3D-QSTR contour maps were utilized to visualize the information on the CoMFA and CoMSIA model properties in three-dimensional space. These maps used characteristics of compounds that are crucial for activity and display the regions around molecules where the variance of activities is expected based on physicochemical property changes in molecules [52]. The analysis of favorable and unfavorable regions of steric, electrostatic, hydrophobic, HBD, and HBA atom fields contribute to the realization of the relationship between the aconitine alkaloid’s toxic activity and its structure. Steric and electrostatic contour maps of the CoMFA QSTR model are shown in Figure 4A,B, respectively. Hydrophobic, HBD, and HBA contour maps of the CoMSIA QSTR model are shown in Figure 4C–E. Compound **6** has the most toxic activity, so it was chosen as the reference structure for the generation of the CoMFA and CoMSIA contour maps.

**Figure 6:**
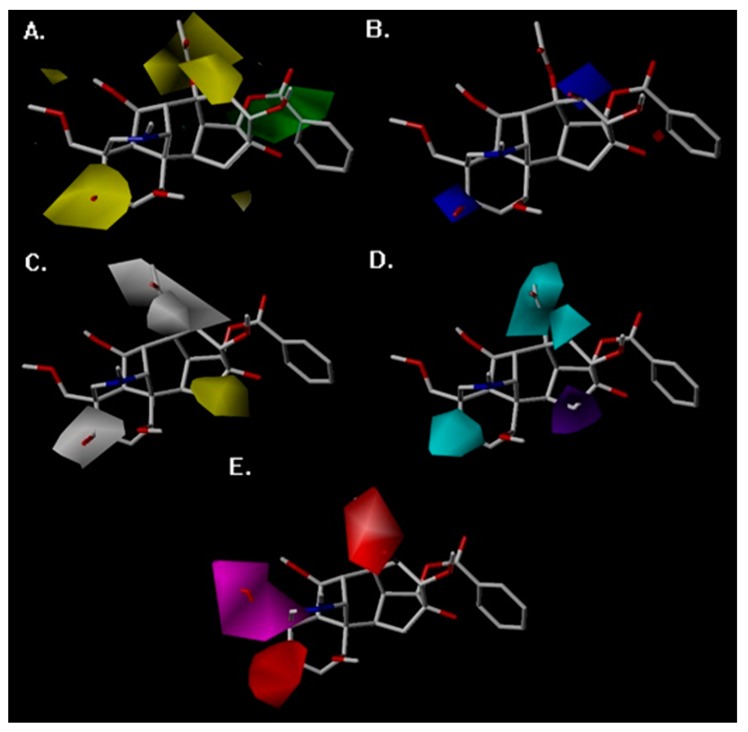
CoMSIA and CoMFA contour maps around Compound **6**. (**A**) The CoMFA steric contour map, (**B**) the CoMFA electrostatic contour map, (**C**) the CoMSIA hydrophobic field contour maps, (**D**) the CoMSIA hydrogen bond donor field, and (**E**) the CoMSIA hydrogen bond acceptor field.

In the case of the CoMFA study, the steric contour map around Compound **6** is shown in Figure 6A. The yellow regions near R2, R7, and R6 showed the substituents of the molecule, which proved that these positions were not ideal for sterically favorable functional groups. Therefore, Compounds **19**, **24**, and **26** (with pLD_50_ values of 1.17, 0.84, and 1.82, respectively), which consist of sterically esterified moieties at Positions R2 and R7, were less toxic than Compounds **6** and **20** (with pLD_50_ values of 5.00 and 4.95), which were substituted by a small hydroxyl group, and Compound **3** (with a pLD_50_ value of 1.44) has less toxic activity due to the esterified moiety in R6. The green regions, sterically favorable charges, were favorable for toxicity and are shown around R9, so the small group substituted in R9 (Compounds **23**, **29**, **30**, and **31**, with pLD_50_ values of 1.85, 2.29, 1.93, and 1.85, respectively) exhibited less toxicity.

The CoMFA electrostatic contour map is shown in Figure 6B. The blue region near the R2 and R7 substitution revealed that the replacement of electropositive groups is in favor of toxicity. This can be proven by the fact that the compounds with hydroxy in these two positions had higher pLD_50_ values than the compound with acetoxy or no substituents. The red region surrounding molecular scaffolds was not distinct, which revealed that there was no connection between the electronegative and the toxicity.

The CoMSIA hydrophobic contour map is shown in Figure 6C. The R2, R6, and R7 around the white region indicated that the hydrophobic groups were unfavorable for the toxicity, so the esterification of hydrophilic hydroxyl or dehydroxylation decreased the toxicity, which is consistent with the steric and electrostatic contour map. The yellow contour map near the R12 manifested that the hydrophilic hydroxy was unfavorable to the toxicity, which can be validated by the fact that aconitine alkaloids with hydroxy substituents in R12 (Compound **10**, with a pLD_50_ value of 1.88) have a lower pLD_50_ value than the compounds with no substitution (Compound **1**, with a pLD_50_ value of 4.92). 

The CoMSIA contour map of HBD is shown in Figure 6D. The cyan regions at R2, R6, and R7 represented a favorable condition for the HBD atom, which clearly validated the fact that the compounds with hydroxy in this region show potent toxicity. A purple region was found near R12, which proved that the HBD atom (hydroxyl) in this region has an adverse effect on toxicity. The HBA contour map is shown in Figure 6. The magenta region around R1 substitution proved that this substitution was favorable to the HBA atom, so Compounds **13**, **15**, **32**, and **33** with the HBA atom in the R1 substitution exhibit more potent toxicity (with pLD_50_ values of 3.52, 3.30, 3.16, and 2.84) than compounds with methoxymethyl substituents (Compounds **19**, **24**, and **26** with pLD_50_ values of 1.17, 0.84, and 1.82). The red contour map where HBA atoms are unfavorable for the toxicity was positioned around R2 and R6. These contours were well validated by the lower pLD_50_ value of compounds with carbonyl in these substitutions.

**Figure 7:**
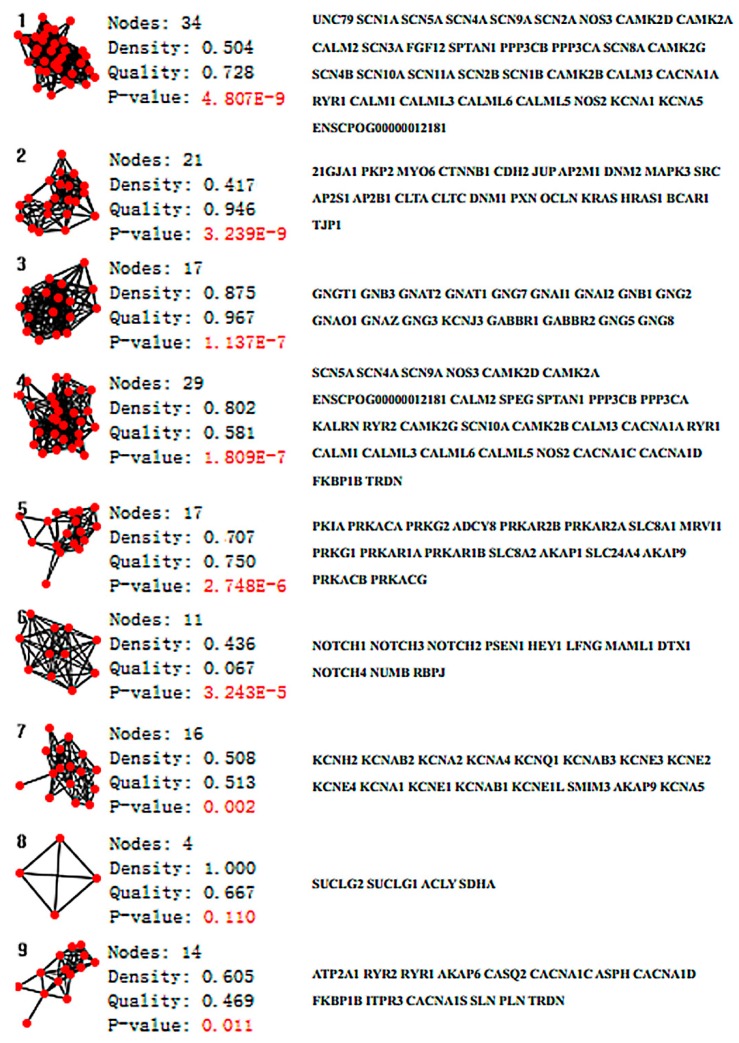
Sub-graphs of the PPI network of aconitine alkaloids induced cardiotoxicity analyzed using the ClusterONE plugin.

The PPI network of aconitine alkaloids cardiotoxicity was divided into nine clusters using ClusterONE. Statistical parameters are shown in Figure 5. Six clusters, namely Clusters 1, 3, 4, 5, 7, and 9, which possess quality scores higher than 0.5, a density higher than 0.45, and a *p*-value less than 0.05, were selected for further analysis (in Figure 7). Clusters 1, 4, and 7 consisted of proteins mainly involved in the effects of various calcium, potassium, and sodium channels. Cluster 1 mainly consisted of three channel types related to the cardiotoxicity of aconitine alkaloids, Cluster 4 contained calcium and sodium channels and some channel exchangers (such as RYR1 and RYR2), and Cluster 7 mainly consisted of various potassium channels. All of these findings are consistent with previous research about the arrhythmogenic properties of the toxicity of aconitine alkaloids: the aconitine binds to ion channels and affects their open state, and thus the corresponding ion influx into the cytosol [53,54,55]. The channel exchangers play a crucial role in keeping the ion transportation and homeostasis inside and outside of the cell. Cluster 9 contained some regulatory proteins that can activate or repress the ion channels through the protein expression level. ATP2A1, RYR2, RYR1, CACNA1C, CACNA1D, and CACNA1S mediate the release of calcium, thereby playing a key role in triggering cardiac muscle contraction and maintaining the calcium homeostasis [56,57]. Aconitine may cause aberrant channel activation and lead to cardiac arrhythmia. Clusters 3 and 5 consisted of cAMP-dependent protein kinase (cAPK), cGMP-dependent protein kinase (cGPK), and guanine nucleotide binding protein (G protein). They have not been fully studied to prove whether the cardiotoxicity induced by aconitine alkaloids is linked to the cAPK, cGPK, and G proteins; however, some studies have shown that cardiotoxicity-related protein KCNJ3 (potassium inwardly-rectifying channel) is controlled by G proteins and the cardiac sodium/calcium exchanger and is said to be regulated by cAPK and cGPK [58,59]. The result of ClusterONE indicated that the constructed network is consistent with existing studies and that the network can be used to screen essential proteins in the CytoNCA plugin.

**Figure 8:**
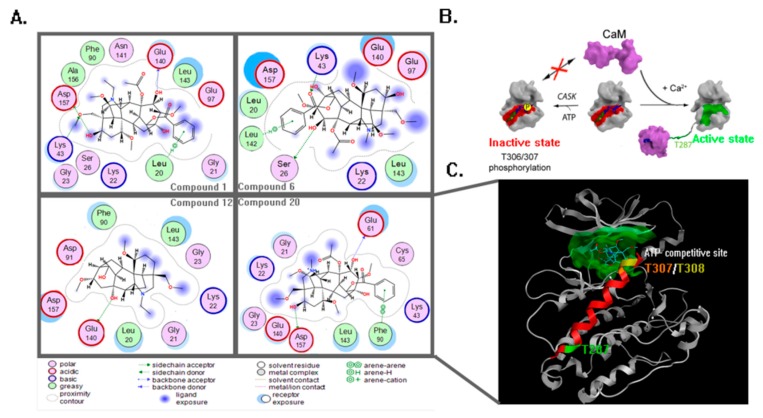
(**A**) The interactions between the four compounds and amino acids are shown by the ligand interaction function in MOE software. (**B**) The mechanisms of the CaMKII activation state and inactivation state. (**C**) The dock result of Compound **20**. Compound **20** docked into 2V7O, and the ATP-competitive pocket was painted green; the T287, T307, and T308 phosphorylation sites were painted green, orange, and yellow, respectively; the inhibitory helix was painted red.

The protein 2V7O belonging to the CaMKII (Calcium/Calmodulin (Ca^2+^/CaM)-dependent serine/threonine kinases II) isozyme protein family plays a central role in cellular signaling by transmitting Ca^2+^ signals. The CaMKII enzymes transmit calcium ion (Ca^2+^) signals released inside the cell by regulating signal transduction pathways through phosphorylation. Ca^2+^ first binds to the small regulatory protein CaM, and this Ca^2+^/CaM complex then binds to and activates the kinase, which then phosphorylates other proteins such as ryanodine receptor and sodium/calcium exchanger. Thus, these proteins are related to the cardiotoxicity induced by aconitine alkaloids [60,61,62]. The excessive activity of CaMKII has been observed in some structural heart disease and arrhythmias [63], and past findings demonstrate neuroprotection in neuronal cultures treated with inhibitors of CaMKII immediately prior to excitotoxic activation of the CaMKII [64]. The acute cardiotoxicity of the aconitine alkaloids is possibly related to this target. Based on the analysis of the PPI network above, CaMKII was selected as the potential target for further molecular docking and dynamic simulation. The dock result of 2V7O is shown in Figure 8A. Compound **20** has the highest fit scores, so it was selected as the template for conformational analysis. The mechanisms of CaMKII activation and inactivation are shown in Figure 8B. Compound **20** affects the normal energy metabolism of the myocardial cell via binding in the ATP-competitive site in Figure 8C. The inactive state of the CaMKII was regulated by CASK-mediated T306/T307 phosphorylation, and this state can be inhibited by the binding of Compound **20** in the ATP-competitive site. Such binding moves CaMKII toward a Ca^2+^/CaM-dependent activation active state and a Ca^2+^/CaM-dependent activation through structural rearrangement of the inhibitory helix caused by Ca^2+^/CaM binding and the subsequent autophosphorylation of T287 [65], which will induce the excessive activity of CaMKII and dynamic imbalance of the calcium ions in the myocardial cell, eventually leading to heart disease and arrhythmias.

The information of a binding pocket of a receptor for its ligand is very important for drug design, particularly for conducting mutagenesis studies [28]. As has been reported in the past [66], the binding pocket of a protein receptor to a ligand is usually defined by those residues that have at least one heavy atom within a distance of 5 Å from a heavy atom of the ligand. Such a criterion was originally used to define the binding pocket of ATP in the Cdk5–Nck5a complex [20], which was later proved to be very useful in identifying functional domains and stimulating the relevant truncation experiments. A similar approach has also been used to define the binding pockets of many other receptor–ligand interactions important for drug design [30,31,33,67,68,69,70]. The information of a binding pocket of CaMKII for the aconitine alkaloids will serve as a guideline for designing drugs with similar scaffolds, particularly for conducting mutagenesis studies.

In Figure 8A, four top fit scores—Compounds **1**, **6**, **12**, and **20**—generated similar significant interactions with amino acid residues around the ATP-competitive binding pocket. Four compounds formed with many Van Der Waals interactions within the noncompetitive inhibitor pocket through amino acid residues such as Asp157, Lys43, Glu140, Lys22, and Leu143. The ligand–receptor interaction showed that the hydroxy in R2 formed a side chain donor interaction with Asp157. In addition, the hydroxy in R6 and R7 also formed a side chain acceptor interaction with Glu140 and Ser26, respectively (the docking result of Compounds **6** and **12** in Figure 8A). These results correspond to the CoMFA and CoMSIA contour maps. However, the small electropositive and hydrophilic group in R2, R6, and R7 possess a certain enhancement function to toxicity.

There were aromatic interactions between the phenyl group in R9 and amino acid residues. The phenyl group in R9 formed aromatic interactions with Leu20, Leu142, and Phe90, while the small group hydroxyl did not form any interaction with Asp91, which demonstrate that bulky phenyl group is crucial to this binding pattern and toxicity. This was mainly equal to the CoMFA steric contour map, where R9 was ideal for sterically favorable groups. The methoxymethyl R1 generated backbone acceptor with Lys43, which correspond to the CoMSIA HBA contour map, where R1 was favorable for the HBA atom. 

**Figure 9:**
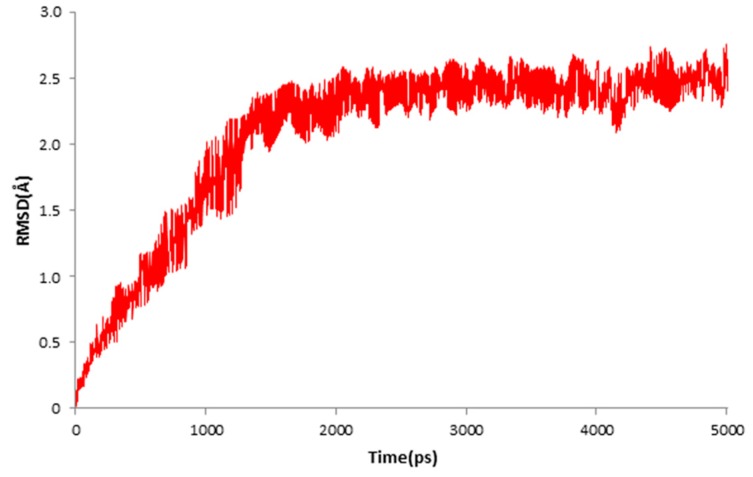
RMSD tendency of the ligand–receptor complex at different MD simulation times in the 5 ns MD simulation.

The result of MD simulation is shown in Figure 9. The red plot represented the RMSD values of the docked protein. The values of RMSD reached 2.41 Å in 1.4 ns and then remained between 2 and 2.5 Å throughout the simulation for up to 5 ns. The averaged value of the RMSD was 2.06 Å. The MD simulation demonstrated that the ligand was stabilized in the active site.

**Figure 10:**
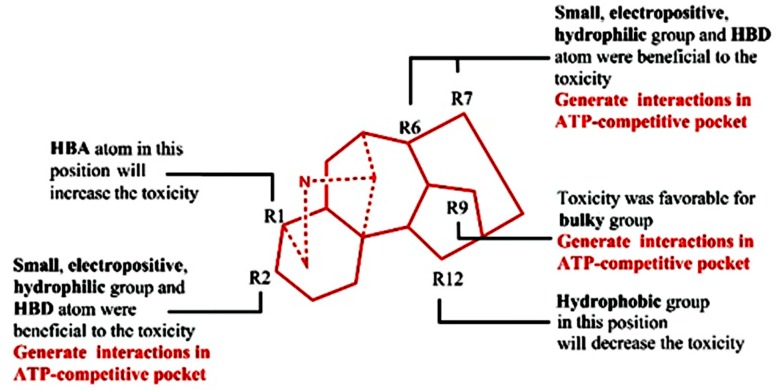
Crucial requirement of cardiotoxicity mechanism was obtained from the ligand-based 3D-QSTR and structure-based molecular docking study.

Finally, we combined the ligand-based 3D-QSTR analysis with the structure-based molecular docking study to identify the necessary moiety related to the cardiotoxicity mechanism of the aconitine alkaloids (in Figure 10). 

## 4. Materials and Methods

### 4.1. Network Analysis

To build the PPI network of aconitine alkaloids, literature from 1 January 2007 to 31 February 2017 was retrieved from PubMed (http://pubmed.cn/) and Web of Science (http://www.isiknowledge.com/) with the MeSH word “aconitine” and “toxicity” and without language restriction. All documents about cardiotoxicity caused by aconitine alkaloids were collected. The proteins related to the aconitine alkaloids cardiotoxicity of this decade were gathered and taken as the input protein in the STRING (https://string-db.org/) database [51,71], used to search for related proteins or pathways that had been reported. Finally, all the proteins and its partners were recorded in Excel in order to import information and build a PPI network in Cytoscape software.

Cytoscape is a free, open-source, Java application for visualizing molecular networks and integrating them with gene expression profiles [71,72]. Plugins are available for network and molecular profiling analyses, new layouts, additional file format support, making connections with databases, and searching within large networks [71].

ClusterONE (Clustering with Overlapping Neighborhood Expansion) of Cytoscape was utilized to cluster the PPI network into overlapping sub-graphs of highly interconnected nodes. ClusterONE is a plugin for detecting and clustering potentially overlapping protein complexes from PPI data. The quality of a group was assessed by the number of sub-graphs, *p*-values, and density. The cluster was discarded when the number of sub-graphs was smaller than 3, the density was less than 0.45, the quality was less than 0.5, and the *p*-value was under 0.05 [73]. The clustering results of the ClusterONE are instrumental to understanding how the reliability of the PPI network relates to aconitine alkaloids’ cardiotoxicity.

CytoNCA is a plugin in Cytoscape integrating calculation, evaluation, and visualization analysis for multiple centrality measures. There are eight centrality measurements provided by CytoNCA: betweenness, closeness, degree, eigenvector, local average connectivity-based, network, subgraph, and information centrality [74]. The primary purpose of the centrality analysis was to confirm the essential proteins in the pre-built PPI network. The three centrality measurements in the CytoNCA plugin—subgraph centrality, betweenness centrality, and closeness centrality—were used for evaluating and screening the essential protein in the merged target network. 

The subgraph centrality characterizes the participation of each node in all subgraphs in a network. Smaller subgraphs are given more weight than larger ones, which makes this measurement an appropriate one for characterizing network properties. The subgraph centrality of node “*u*” can be calculated by [75]

$$C_{S}\left(u\right)=\sum_{l=0}^{\infty }\frac{\mu_{l}\left(u\right)}{l!}=\sum_{v=1}^{N}{\left(v_{v}^{u}\right)}^{2}e^{\lambda v}.$$

$\mu_{l}\left(u\right)$ is the uth diagonal entry of the lth power of the weight adjacency matrix of the network. $v_{1}$, $v_{2}$, …, $v_{N}$ is be an orthonormal basis composed of $R^{N}$ composed by eigenvectors of A associated to the eigenvalues $\lambda_{1}$, $\lambda_{2}$, …, $\lambda_{N}v_{v}^{u}$, which is the *u*th component of $v_{v}$ [75].

The betweenness centrality finds a wide range of applications in network theory. It represents the degree to which nodes stand between each other. Betweenness centrality was devised as a general measure of centrality. It is applicable to a wide range of problems in network theory, including problems related to social networks, biology, transport, and scientific cooperation. The betweenness centrality of a node u can be calculated by [76]

$$C_{B}\left(u\right)=\underset{s\neq u\neq t}{\sum }\frac{\rho \left(s,u,t\right)}{\rho \left(s,t\right)}.$$

*ρ* (*s*, *t*) is the total number of shortest paths from node s to node *ρ*; (*s*, *u*, *t*), which is the number of those paths that pass through *u*.

Closeness centrality of a node is a measure of centrality in a network, calculated as the sum of the length of the shortest paths between the node and all other nodes in the graph. Thus, the more central a node is, the closer it is to all other nodes. The closeness centrality of a node *u* can be calculated by [77]

$$C_{C}\left(u\right)=\frac{\left|N_{U}\right|-1}{\sum_{V}dist\left(u,v\right)}.$$

|*Nu*| is the number of node *u*’s neighbors, and *dist (u, v)* is the distance of the shortest path from node *u* to node *v*.

PharmMapper serves as a valuable tool for identifying potential targets for a novel synthetic compound, a newly isolated natural product, a compound with known biological activity, or an existing drug [78]. Of all the aconitine alkaloids in this research, Compounds **6**, **12**, and **20** exhibited the most toxic activity and were used for the potential target prediction. The Mol2 format of three compounds was submitted to the PharmMapper server. The parameters of Generate Conformers and Maximum Generated Conformations was set as ON and 300, respectively. Other parameters used default values. Finally, the result of the ClusterONE and PharmMapper will be combined together to select the potential targets for the following docking study [78].

### 4.2. QSTR Research

Comparative molecular field analysis (CoMFA) and comparative molecular similarity index analysis (CoMSIA) are efficient tools in ligand-based drug design and are in use for contour map generation and identification of favorable and unfavorable regions in a moiety [52,79]. The CoMFA consists of a steric and electrostatic contour map of molecules that are correlated with toxic activity, while the CoMSIA consists of hydrophobic field, hydrogen bond donor (HBD)/hydrogen bond acceptor (HBA) [80], and steric/electrostatic fields that are correlated with toxic activity. The CoMFA and CoMSIA have been utilized to generate a 3D-QSTR model [81]. All molecule models and the generation of 3D-QSTR were performed with SYBYL X2.0. Alkaloids in mice with LD_50_ values listed in Table 4 were extracted from recent literature [70]. The LD_50_ values of all aconitine alkaloids were converted into pLD_50_ with a standard TRIPOS force field. These pLD50 values were used as a dependent variable, while CoMFA and CoMSIA descriptors were used as an independent variable. The SKETCH function of Sybyl X2.0 was utilized to illustrate structure and charges, and was calculated by the Gasteiger–Huckel method. Additionally, the tripose force field was utilized for energy minimization of these aconitine alkaloid molecules [81]. The 31 molecules were divided into a ratio of 3:1. The division was done in a way that showed that both datasets are balanced and consist of both active and less active molecules [81]. The reliability of the 3D-QSTR model depends on the database molecular alignment. The most toxic aconitine alkaloids (Compound **6**) was selected as the template molecule, and the tetradecahydro-2*H*-3,6,12-(epiethane [1,1,2] triyl)-7,9-methanonaphtho[2,3-*b*]azocine was selected as the common moiety.

PLS (partial least squares) techniques are associated with field descriptors with activity values such as [80] Leave One Out (LOO) values, the optimal number of components, the Standard Error of Estimation (SEE), cross-validated coefficients (q^2^), and the conventional coefficient (r^2^). These statistical data are pivotal in the evaluation of the 3D-QSTR model and can be worked out in the PLS method [81]. The model is said to be good when the q^2^ value is more than 0.5 and the r^2^ value is more than 0.6. The q^2^ and r^2^ values reflect a model’s soundness. The best model has the highest q^2^ and r^2^ values, the lowest SEE, and an optimal number of components [80,82,83]. In the case of CoMFA and CoMSIA analysis, the values of the optimal number of components, SEE, and q^2^ can be worked out by LOO validation, with USE SAMPLS turned on and components set to 5, while in the process of calculating r^2^, the USE SAMPLS was turned off and the column filtration was set to 2.0 kcal mol^−1^ in order to speed up the calculation without the need to sacrifice information content [81,82,83,84]. Therefore, components were set to 6 and 4, respectively, which were optimal numbers of components calculated by performing a SAMPLS run. SEE and r^2^ were utilized to assess the non-cross validated model.

The Applicability Domain (AD) of the Topomer CoMFA and CoMSIA model was confirmed by the Williams plot of Residuals vs. Leverage. Leverage of a query chemical is proportional to its Mahalanobis distance measure from the centroid of the training set [85,86]. The leverages are calculated for a given dataset X by obtaining the leverage matrix (H) with the equation below:

$$H=X{\left(X^{T}X\right)}^{-1}X^{T}.$$

X is the model matrix, while XT is its transpose matrix. The plot of standardized residuals vs. leverage values was drawn, and compounds with standardized residuals greater than three standard deviation units (±3σ) were considered as outliers [85]. The critical leverage value is considered 3 P/n, where p is the number of model variables plus one, and n is the number of objects used to calculate the model. h > 3 P/n mean predicted response is not acceptable [85,86,87].

### 4.3. Molecular Docking and Dynamics Study

MOE (Molecular Operating Environment) is a comprehensive Computer-Aided Drug Design (CADD) software program that incorporates the functions of QSAR, molecular docking, molecular dynamics, ADME (absorption, distribution, metabolism, and excretion), and homologous modeling. All of these functions are regarded as conducive instruments in the field of drug discovery and biochemistry. The molecular docking and dynamics technology were performed in MOE2016 software to detect the stability and affinity between the ligands and predictive targets [88,89].

The docking process involves the prediction of ligand conformation and orientation within a targeted binding site. Docking analysis is an important step in the docking process. It has been widely used to study the reasonable binding mode and obtain information of interactions between amino acids in active protein sites and ligands. The molecular docking analysis was carried out to determine the toxicity-related moiety of aconitine alkaloids through the ligand–amino-acid interaction function in MOE2015. The PDB format of 2V7O and 2VZ6 was downloaded from the PDB (protein data bank) database (https://www.rcsb.org/), and the mol2 format of compounds was from the SYBYL software of QSTR research. The structure preparation function in MOE software will be carried out to minimize the energy and optimize the structure of the protein skeleton. Based on the London dG score and induced fit refinement, all compounds will be docked into the active site of every potential target by taking score values as the scoring function [90].

The DCG (Discounted Cumulative Gain) algorithm was utilized to examine the consistency between the ranking result of pLD_50_ and our research (fit scores of dock study). They rely on the formula that refers to pLD_50_. The IDCG (ideal DCG) refers to the ordered pLD_50_ values. The closer the Normalized Discounted Cumulative Gain (NDCG) value is to 1, the better the consistency [91].

$$NDCG_{P}=\frac{DCG_{P}}{IDCG_{P}}\;DCG_{P}=rel_{1}+\overset{p}{\underset{i=2}{\sum }}\frac{rel_{i}}{{log}_{2}i}.$$

Preliminary MD simulations for the model protein were performed using the program NAMD (NAnoscale Molecular Dynamics program, v 2.9), and all files were generated using visual molecular dynamics (VMD). NAMD is a freely available software designed for high-performance simulation of large biomolecular systems [92]. During the MD simulation, minimization and equilibration of original and docked proteins occurred in a 15 Å3 size water box. A CHARMM 22 force field file was applied for energy minimization and equilibration with Gasteiger–Huckel charges using Boltzmann initial velocity [93,94]. Integrator parameters also included 2 fs/step for all rigid bonds and nonbonded frequencies were selected for 1 Å and full electrostatic evaluations for 2 Å were used with 10 steps for each cycle [93]. The particle mesh Ewald method was used for electrostatic interactions of the simulation system periodic boundary conditions with grid dimensions of 1.0 Å [94]. The pressure was maintained at 101.325 kPa using the Langevin piston and the temperature was controlled at 310 K using Langevin dynamics. Covalent interactions between hydrogen and heavy atoms were constrained using the SHAKE/RATTLE algorithm. Finally, 5 ns MD simulations for original and docked protein were carried out for comparing and verifying the binding affinity and stability of the ligand–receptor complex.

## 5. Conclusions

The method combining network analysis and the in silico method was carried out to illustrate the QSTR and toxic mechanisms of aconitine alkaloids. The 3D-QSTR was built in Sybyl with internal robustness and external high prediction, enabling identification of pivotal molecule moieties related to toxicity in aconitine alkaloids. The CoMFA model had q^2^, r^2^, optimum component, and correlation coefficient (r^2^) values of 0.624, 0.966, 6, and 0. 9698, respectively, and the CoMSIA model had q^2^, r^2^, optimum component, and correlation coefficient (r^2^) values of 0.719, 0.901, 4, and 0.9770. The network was built with Cytoscape software and the STRING database, which demonstrated the reliability of cluster analysis. The 2V7O and 2VZ6 proteins were identified as potential targets with the CytoNCA plugin with PharmMapper server for interactions between the aconitine alkaloids and key amino acids in the dock study. The result of the dock study demonstrates the consistency of the experimental pLD_50_. The MD simulation indicated that aconitine alkaloids exhibit potent binding affinity and stability to the receptor CAMK2G. Finally, we incorporate pivotal molecule moieties and ligand–receptor interactions to realize the QSTR of aconitine alkaloids. This research serves as a guideline for studies of toxicity, including neuro-, reproductive, and embryo-toxicity. With a deep understanding of the relationship between toxicity and structure of aconitine alkaloids, subsequent structural modification of aconitine alkaloids can be carried out to enhance their efficacy and to reduce their toxic side effects. Based on such research, aconitine alkaloids can bring us closer to medical and clinical applications. In addition, as pointed out in past research [95], user-friendly and publicly accessible web servers represent the future direction of reporting various important computational analyses and findings [96,97,98,99,100,101,102,103,104,105,106,107,108,109]. They have significantly enhanced the impacts of computational biology on medical science [110,111]. The research in this paper will serve as a foundation for constructing web servers for QSTR studies and target identifications of compounds.

## Figures and Tables

**Table 1:** The partial least square (PLS) statistical parameters for the CoMFA and CoMSIA.

PLS Statistical Parameters	CoMFA	CoMSIA
q^2^ ^a^	0.624	0.719
r^2^ ^b^	0.966	0.901
ONC ^c^	6	4
SEE ^d^	0.043	0.116
F ^e^	124.127	157.458
r^pred2^ ^f^	0.903	0.894
Fraction of Field contribution ^g^		
steric	0.621	0.120
Electrostatic	0.379	0.204
Hydrophobic	-	0.327
H-bond acceptor	-	0.216
H-bond donor	-	0.133

^a^ Cross-validated correlation coefficient; ^b^ Non-cross-validated correlation coefficient; ^c^ Optimum number of components; ^d^ Standard error of estimate; ^e^ F-test value; ^f^ The predictive r^2^ value; ^g^ Field: steric, electrostatic, hydrophobic, hydrogen-bond acceptor, and hydrogen-bond donor.

**Table 2:** Proteins related to aconitine alkaloids induced cardiotoxicity extracted from 274 articles.

Name	Classification	Frequency
RYR2	Ryanodine receptor 2	19
RYR1	Ryanodine receptor 1	15
GJA1	Gap junction α-1 protein (connexin43)	13
SLC8A1	Sodium/calcium exchanger 1	11
ATP2A1	Calcium transporting ATPase fast twitch 1	9
KCNH2	Potassium voltage-gated channel H2	7
SCN3A	Sodium voltage-gated channel type 3,	3
SCN2A	Sodium voltage-gated channel type 2	3
SCN8A	Sodium voltage-gated channel type 8	2
SCN1A	Sodium voltage-gated channel type 1	2
SCN4A	Sodium voltage-gated channel type 4	1
KCNJ3	Potassium inwardly-rectifying channel J3	1

**Table 3:** Ranking results by experimental and predicted pLD_50_ and fit score.

Compounds	Experimental pLD_50_	Fit Score (2V7O)	Fit Score (2VZ6)
6	1	3	3
20	2	1	12
12	3	4	9
1	4	2	4
11	5	7	2
14	6	8	13
16	7	5	6
7	8	17	15
8	9	10	11
27	10	23	17
13	11	12	19
15	12	11	5
32	13	18	18
5	14	22	8
33	15	13	29
21	16	15	1
25	17	9	20
22	18	25	25
17	19	20	16
28	20	24	30
9	21	16	32
29	22	32	14
2	23	30	24
30	24	31	26
18	25	21	27
10	26	26	21
23	27	29	31
31	28	33	7
26	29	14	23
4	30	28	33
3	31	6	10
19	32	27	28
24	33	19	22
NDCG	1	0.9122	0.8503

**Table 4:**
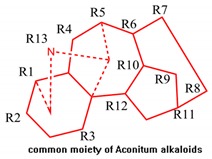
Structure of Aconitine alkaloids with toxic activity.

No.	CAS. NO	Substituent in R1 to R13	pLD_50_
1	302-27-2	methoxymethyl-hydroxy-methoxy-methoxy-H-acetoxyl-hydroxy-methoxy-benzoxy-H-hydroxy-H-ethyl	4.92
2*	545-56-2	methoxymethyl-H-hydroxy-methoxy-hydroxy-hydroxy-H-methoxy-hydroxy-H-H-H-ethyl	1.96
3	127-29-7	methoxymethyl-hydroxy-methoxy-methoxy-H-acetoxyl-H-methoxy-methyl 2,3-dimethoxybenzoate-H-hydroxy-H-ethyl	1.44
4	509-18-2	methoxymethyl-H-hydroxy-methoxy-hydroxy-hydroxy-H-methoxy-methoxy-H-H-H-ethyl	1.76
5*	466-24-0	methoxymethyl-hydroxy-methoxy-methoxy-H-hydroxy-hydroxy-methoxy-benzoxy-H-hydroxy-H-ethyl	3.00
6	2752-64-9	methoxymethyl-hydroxy-methoxy-methoxy-H-acetoxyl-hydroxy-methoxy-benzoxy-H-hydroxy-H-methy	5.00
7	4491-19-4	methoxymethyl-hydroxy-methoxy-methoxy-H-acetoxyl-H-methoxy-benzoxy-H-hydroxy-H-ethyl	4.33
8*	6900-87-4	methoxymethyl-H-methoxy-methoxy-H-acetoxyl-hydroxy-methoxy-benzoxy-H-hydroxy-H-methy	4.33
9	1356-52-1	H-H-methoxy-H-H-hydroxy-H-methoxy-benzoxy-H-H-hydroxy-H-ethyl	2.55
10	6836-11-9	methy-H-methoxy-acetoxyl-dioxolane-H-H-methoxy-methoxy-H-H-hydroxy-ethyl	1.88
11	8006-38-0	methoxymethyl-hydroxy-methoxy-methoxy-H-acetoxyl-hydroxy-methoxy-benzoxy-H-hydroxy-H-ethyl	4.78
12*	20501-56-8	methoxymethyl-H-methoxy-H-H-hydroxy-H-methoxy-hydroxy-H-H-H-ethyl	4.94
13	21019-30-7	2-(3-methyl-2,5-dioxopyrrolidin-1-yl)benzoate ethyl-H-methoxy-methoxy-hydroxy-hydroxy-H-methoxy-methoxy-H-H-H-ethyl	3.52
14	41849-35-8	methoxymethyl-hydroxy-methoxy-methoxy-H-acetoxyl-hydroxy-methoxy-benzoxy-H-hydroxy-hydroxy-ethyl	4.66
15	26000-16-8	2-(3-methyl-2,5-dioxopyrrolidin-1-yl)benzoate ethyl-H-methoxy-methoxy-a-H-H-methoxy-methoxy-H-H-H-ethyl	3.3
16	77181-26-1	methoxymethyl-acetoxyl-methoxy-methoxy-H-acetoxyl-hydroxy-methoxy-benzoxy-H-hydroxy-H-ethyl	4.4
17	71402-60-3	methoxymethyl-hydroxy-methoxy-methoxy-H-hydroxy-hydroxy-methoxy-benzoxy-H-hydroxy-H-trimethylethanaminium	2.59
18	67806-02-4	methoxymethyl-acetoxyl-methoxy-methoxy-H-acetoxyl-acetoxyl-methoxy-benzoxy-H-acetoxyl-H-ethyl	1.9
19	85031-25-0	methoxymethyl-acetoxyl-methoxy-methoxy-H-acetoxyl-acetoxyl-methoxy-acetoxyl-H-acetoxyl-H-ethyl	1.17
20	71425-64-4	methoxymethyl-hydroxy-methoxy-methoxy-H-acetoxyl-hydroxy-methoxy-benzoxy-H-hydroxy-H-trimethylethanaminium	4.95
21*	63238-67-5	methoxymethyl-hydroxy-methoxy-methoxy-H-hydroxy-hydroxy-methoxy-benzoxy-H-hydroxy-H-methy	2.68
22	71402-61-4	methoxymethyl-hydroxy-methoxy-methoxy-H-hydroxy-hydroxy-methoxy-benzoxy-hydroxy-H-H-trimethylethanaminium	2.62
23	38146-89-3	methoxymethyl-hydroxy-methoxy-methoxy-H-hydroxy-H-methoxy-hydroxy-H-hydroxy-H-ethyl	1.85
24	82144-73-8	methoxymethyl-acetoxyl-methoxy-methoxy-H-acetoxyl-H-methoxy-benzoxy-H-hydroxy-H-ethyl	0.84
25	82144-74-9	methoxymethyl-acetoxyl-methoxy-methoxy-H-acetoxyl-H-methoxy-benzoxy-H-acetoxyl-H-ethyl	2.66
26	38146-91-7	methoxymethyl-acetoxyl-methoxy-methoxy-H-acetoxyl-H-methoxy-acetoxyl-H-acetoxyl-H-ethyl	1.82
27*	71402-59-0	methoxymethyl-H-methoxy-methoxy-H-acetoxyl-hydroxy-methoxy-benzoxy-H-hydroxy-H-ethyl	4.27
28	71402-62-5	methoxymethyl-H-hydroxy-methoxy-H-hydroxy-hydroxy-methoxy-benzoxy-H-hydroxy-H-methy	2.59
29	39089-30-0	methy-H-hydroxy-H-H-hydroxy-H-methoxy-hydroxy-H-H-H-ethyl	2.29
30	58111-33-4	methoxymethyl-H-hydroxy-methoxy-hydroxy-hydroxy-H-methoxy-H-methoxy-H-H-trimethylethanaminium	1.93
31*	23943-93-3	hydroxy-H-methoxy-hydroxy-H-hydroxy-H-methoxy-methoxy-H-H-H-ethyl	1.85
32	32854-75-4	2-acetamidobenzoate ethyl-H-methoxy-H-H-hydroxy-H-methoxy-methoxy-hydroxy-H-H-ethyl	3.16
33	138729-51-8	2-acetamidobenzoate ethyl-H-methoxy-H-H-acetoxyl-H-methoxy-methoxy-acetoxyl-H-H-ethyl	2.84

* Test set compound.

## Supplementary Materials

The following are available online, Table S1: The centrality measurements of 147 nodes were calculated by CytoNCA, Table S2: The dock result of the aconitine alkaloids to the proteins 2V7O and 2VZ6.
