# Supplementary material for: A Comprehensive In Silico Method to Study the QSTR of the Aconitine Alkaloids for Designing Novel Drugs
Source: Molecules. 2018 Sep 18;23(9):2385. doi: 10.3390/molecules23092385 (PMC6225272; doi:10.3390/molecules23092385)
Supplement: Supplementary file 1 [file molecules-23-02385-s001.zip › molecules-354597-supplementary/Table S1.pdf]

Table S1. The centrality measurements of 147 nodes were calculated by CytoNCA

Protein	Subgragh	Degree	Eigenvector	Information	LAC	Betweenness	Closeness	Network
UNC79	Subgragh: 2089588.9	Degree: 7.0	Eigenvector: 0.07232144	Information: 4.078461	LAC: 6.571429	Betweenness: 0.0	Closeness: 0.3056133	Network: 7.0
TRH	Subgragh: 24.627142	Degree: 3.0	Eigenvector: 1.7988276E-4	Information: 2.7136943	LAC: 2.0	Betweenness: 0.0	Closeness: 0.24747474	Network: 3.0
TRDN	Subgragh: 1781333.5	Degree: 12.0	Eigenvector: 0.06675436	Information: 5.056548	LAC: 6.6666665	Betweenness: 79.470894	Closeness: 0.3310811	Network: 8.495022
TLX2	Subgragh: 129.83693	Degree: 1.0	Eigenvector: 5.55129E-4	Information: 1.6256909	LAC: 0.0	Betweenness: 0.0	Closeness: 0.26110125	Network: 0.0
TJP1	Subgragh: 3406.994	Degree: 11.0	Eigenvector: 3.752259E-4	Information: 4.8998713	LAC: 7.818182	Betweenness: 75.21795	Closeness: 0.27683616	Network: 9.330556
SUCLG2	Subgragh: 336.19208	Degree: 4.0	Eigenvector: 1.6575721E-4	Information: 3.1330578	LAC: 1.5	Betweenness: 785.5439	Closeness: 0.25880283	Network: 2.6666667
SUCLG1	Subgragh: 1904.4441	Degree: 5.0	Eigenvector: 2.9481309E-5	Information: 3.4929106	LAC: 1.6	Betweenness: 52.727272	Closeness: 0.21273516	Network: 3.1666667
SRC	Subgragh: 4463.0786	Degree: 13.0	Eigenvector: 3.992194E-4	Information: 5.1990433	LAC: 8.153846	Betweenness: 189.98155	Closeness: 0.27788278	Network: 10.753283
SPTR	Subgragh: 22.947414	Degree: 3.0	Eigenvector: 1.7890391E-4	Information: 2.7136943	LAC: 2.0	Betweenness: 0.0	Closeness: 0.24747474	Network: 3.0
SPTAN1	Subgragh: 1.7554642E7	Degree: 27.0	Eigenvector: 0.20962904	Information: 6.36491	LAC: 15.0	Betweenness: 146.27417	Closeness: 0.3245033	Network: 20.600204
SPEG	Subgragh: 453844.44	Degree: 5.0	Eigenvector: 0.033703953	Information: 3.4929101	LAC: 4.0	Betweenness: 0.0	Closeness: 0.27579737	Network: 5.0
SMIM3	Subgragh: 5671.4917	Degree: 1.0	Eigenvector: 0.0037591923	Information: 1.6256909	LAC: 0.0	Betweenness: 0.0	Closeness: 0.25	Network: 0.0
SLN	Subgragh: 1597.9031	Degree: 2.0	Eigenvector: 0.0019852547	Information: 2.2187285	LAC: 1.0	Betweenness: 0.0	Closeness: 0.26873857	Network: 2.0
SLC8A2	Subgragh: 479.4813	Degree: 4.0	Eigenvector: 0.0010086364	Information: 3.1330578	LAC: 2.0	Betweenness: 6.428571	Closeness: 0.2658228	Network: 3.3333333
SLC8A1	Subgragh: 48875.285	Degree: 11.0	Eigenvector: 0.011002112	Information: 4.8998713	LAC: 1.2727273	Betweenness: 3227.5715	Closeness: 0.352518	Network: 3.0444446
SLC24A4	Subgragh: 159.0656	Degree: 2.0	Eigenvector: 6.058204E-4	Information: 2.2187285	LAC: 1.0	Betweenness: 0.0	Closeness: 0.26156583	Network: 2.0
SLC24A1	Subgragh: 129.83714	Degree: 1.0	Eigenvector: 5.55129E-4	Information: 1.6256909	LAC: 0.0	Betweenness: 0.0	Closeness: 0.26110125	Network: 0.0
SDHA	Subgragh: 26.816303	Degree: 3.0	Eigenvector: 1.03786915E-5	Information: 2.7136943	LAC: 2.0	Betweenness: 0.0	Closeness: 0.20851064	Network: 3.0
SCN9A	Subgragh: 7722179.0	Degree: 14.0	Eigenvector: 0.13903475	Information: 5.329195	LAC: 12.571428	Betweenness: 4.111687	Closeness: 0.31612903	Network: 13.0
SCN8A	Subgragh: 1.648715E7	Degree: 27.0	Eigenvector: 0.2031519	Information: 6.36491	LAC: 14.333333	Betweenness: 699.464	Closeness: 0.3888889	Network: 19.604874
SCN5A	Subgragh: 1.8514544E7	Degree: 31.0	Eigenvector: 0.21528004	Information: 6.5484676	LAC: 14.451612	Betweenness: 210.63019	Closeness: 0.34186047	Network: 22.573725
SCN4B	Subgragh: 8115817.5	Degree: 15.0	Eigenvector: 0.14253327	Information: 5.4485474	LAC: 12.8	Betweenness: 4.2251296	Closeness: 0.31545064	Network: 12.857142
SCN4A	Subgragh: 1.2874699E7	Degree: 22.0	Eigenvector: 0.17952275	Information: 6.0690484	LAC: 14.909091	Betweenness: 471.72296	Closeness: 0.380829	Network: 17.536987
SCN3A	Subgragh: 1.1230335E7	Degree: 22.0	Eigenvector: 0.16766313	Information: 6.0690484	LAC: 12.636364	Betweenness: 458.6836	Closeness: 0.383812	Network: 14.942498
SCN2B	Subgragh: 4642965.5	Degree: 11.0	Eigenvector: 0.10780419	Information: 4.8998713	LAC: 10.909091	Betweenness: 0.0	Closeness: 0.31012657	Network: 11.0
SCN2A	Subgragh: 1.5761408E7	Degree: 25.0	Eigenvector: 0.19863097	Information: 6.256977	LAC: 14.4	Betweenness: 582.7256	Closeness: 0.3878628	Network: 18.782938
SCN1B	Subgragh: 8115817.5	Degree: 15.0	Eigenvector: 0.14253327	Information: 5.4485474	LAC: 12.8	Betweenness: 4.2251296	Closeness: 0.31545064	Network: 12.857142
SCN1A	Subgragh: 1.576141E7	Degree: 25.0	Eigenvector: 0.19863099	Information: 6.256977	LAC: 14.4	Betweenness: 582.7256	Closeness: 0.3878628	Network: 18.782938
SCN11A	Subgragh: 1.2825613E7	Degree: 23.0	Eigenvector: 0.1791823	Information: 6.1355953	LAC: 11.130435	Betweenness: 166.26895	Closeness: 0.3402778	Network: 14.024
SCN10A	Subgragh: 2.1285332E7	Degree: 30.0	Eigenvector: 0.2308326	Information: 6.5061	LAC: 14.7	Betweenness: 136.02132	Closeness: 0.32885906	Network: 22.48266
RYR2	Subgragh: 5008254.0	Degree: 21.0	Eigenvector: 0.1119402	Information: 5.998078	LAC: 8.380953	Betweenness: 2565.1418	Closeness: 0.4072022	Network: 13.886735
RYR1	Subgragh: 3507420.0	Degree: 18.0	Eigenvector: 0.093674414	Information: 5.753736	LAC: 7.111111	Betweenness: 960.59735	Closeness: 0.35507247	Network: 10.331071
RYA-R44F	Subgragh: 12.946826	Degree: 1.0	Eigenvector: 1.5892624E-4	Information: 1.6256909	LAC: 0.0	Betweenness: 0.0	Closeness: 0.24664429	Network: 0.0
RBPJ	Subgragh: 1554.7837	Degree: 10.0	Eigenvector: 0.0011919751	Information: 4.7267814	LAC: 7.2	Betweenness: 30.74667	Closeness: 0.256993	Network: 9.541667
PXN	Subgragh: 2801.8445	Degree: 10.0	Eigenvector: 2.053579E-4	Information: 4.7267814	LAC: 7.0	Betweenness: 18.694143	Closeness: 0.23863636	Network: 8.333333
PU	Subgragh: 50.14162	Degree: 7.0	Eigenvector: 2.1539956E-4	Information: 4.078461	LAC: 3.142857	Betweenness: 9.333333	Closeness: 0.24915254	Network: 7.0
PSEN1	Subgragh: 49802.414	Degree: 10.0	Eigenvector: 0.011045195	Information: 4.7267814	LAC: 5.2	Betweenness: 1489.8735	Closeness: 0.33333334	Network: 6.2083335
PRKG2	Subgragh: 7886.028	Degree: 10.0	Eigenvector: 0.004205602	Information: 4.7267814	LAC: 4.0	Betweenness: 293.62674	Closeness: 0.27071825	Network: 5.986111
PRKG1	Subgragh: 7886.028	Degree: 10.0	Eigenvector: 0.004205602	Information: 4.7267814	LAC: 4.0	Betweenness: 293.62674	Closeness: 0.27071825	Network: 5.986111
PRKAR2B	Subgragh: 75163.56	Degree: 11.0	Eigenvector: 0.013537675	Information: 4.8998713	LAC: 7.4545455	Betweenness: 66.12146	Closeness: 0.26156583	Network: 9.011111
PRKAR2A	Subgragh: 75163.56	Degree: 11.0	Eigenvector: 0.013537675	Information: 4.8998713	LAC: 7.4545455	Betweenness: 66.12146	Closeness: 0.26156583	Network: 9.011111
PRKAR1B	Subgragh: 48654.85	Degree: 10.0	Eigenvector: 0.010850257	Information: 4.7267814	LAC: 7.2	Betweenness: 38.44781	Closeness: 0.25971732	Network: 8.301587
PRKAR1A	Subgragh: 48654.85	Degree: 10.0	Eigenvector: 0.010850257	Information: 4.7267814	LAC: 7.2	Betweenness: 38.44781	Closeness: 0.25971732	Network: 8.301587
PRKACG	Subgragh: 947111.5	Degree: 15.0	Eigenvector: 0.04861813	Information: 5.4485474	LAC: 7.2	Betweenness: 722.616	Closeness: 0.33870968	Network: 10.069841
PRKACB	Subgragh: 947111.6	Degree: 15.0	Eigenvector: 0.048618134	Information: 5.4485474	LAC: 7.2	Betweenness: 722.616	Closeness: 0.33870968	Network: 10.069841
PRKACA	Subgragh: 947111.5	Degree: 15.0	Eigenvector: 0.04861813	Information: 5.4485474	LAC: 7.2	Betweenness: 722.616	Closeness: 0.33870968	Network: 10.069841
PPP3CB	Subgragh: 2436366.2	Degree: 11.0	Eigenvector: 0.078091055	Information: 4.8998713	LAC: 7.5454545	Betweenness: 7.0653596	Closeness: 0.27946767	Network: 8.9
PPP3CA	Subgragh: 6763875.5	Degree: 21.0	Eigenvector: 0.13010082	Information: 5.998078	LAC: 10.095238	Betweenness: 153.71782	Closeness: 0.33182845	Network: 14.072189
PLN	Subgragh: 148687.6	Degree: 6.0	Eigenvector: 0.019265676	Information: 3.8050835	LAC: 3.3333333	Betweenness: 64.4666	Closeness: 0.30184805	Network: 4.8
PLE	Subgragh: 31.884657	Degree: 4.0	Eigenvector: 1.8948228E-4	Information: 3.1330576	LAC: 3.0	Betweenness: 0.0	Closeness: 0.24789207	Network: 4.0
PKP2	Subgragh: 1109.8438	Degree: 6.0	Eigenvector: 3.0873663E-4	Information: 3.8050835	LAC: 4.3333335	Betweenness: 1.0	Closeness: 0.27222222	Network: 5.2

PKIA	Subgragh: 24641.607	Degree: 5.0	Eigenvector: 0.007784899	Information: 3.4929101	LAC: 1.6	Betweenness: 36.298462	Closeness: 0.25744307	Network: 2.0
PCD	Subgragh: 37.85773	Degree: 5.0	Eigenvector: 1.9815318E-4	Information: 3.4929106	LAC: 3.2	Betweenness: 1.3333334	Closeness: 0.2483108	Network: 5.0
OCLN	Subgragh: 2397.6755	Degree: 9.0	Eigenvector: 3.4593165E-4	Information: 4.534564	LAC: 5.7777777	Betweenness: 89.35479	Closeness: 0.27579737	Network: 6.5
NUMB	Subgragh: 1571.4392	Degree: 9.0	Eigenvector: 0.0012054602	Information: 4.534564	LAC: 6.6666665	Betweenness: 44.934574	Closeness: 0.2658228	Network: 7.839286
NOTCH4	Subgragh: 2980.1252	Degree: 11.0	Eigenvector: 0.0021594414	Information: 4.8998713	LAC: 7.090909	Betweenness: 229.22473	Closeness: 0.2859922	Network: 9.841666
NOTCH3	Subgragh: 3369.3237	Degree: 12.0	Eigenvector: 0.0022225315	Information: 5.056548	LAC: 7.1666665	Betweenness: 338.52066	Closeness: 0.29817444	Network: 10.414142
NOTCH2	Subgragh: 3369.3242	Degree: 12.0	Eigenvector: 0.0022225315	Information: 5.056548	LAC: 7.1666665	Betweenness: 338.52066	Closeness: 0.29817444	Network: 10.414142
NOTCH1	Subgragh: 3369.3242	Degree: 12.0	Eigenvector: 0.0022225315	Information: 5.056548	LAC: 7.1666665	Betweenness: 338.52066	Closeness: 0.29817444	Network: 10.414142
NOS3	Subgragh: 1.6134919E7	Degree: 25.0	Eigenvector: 0.20097198	Information: 6.256977	LAC: 14.24	Betweenness: 151.52411	Closeness: 0.3394919	Network: 17.966318
NOS2	Subgragh: 1749140.0	Degree: 8.0	Eigenvector: 0.066165365	Information: 4.3198524	LAC: 5.5	Betweenness: 1.0	Closeness: 0.27893737	Network: 6.285714
MYO6	Subgragh: 1420.0408	Degree: 8.0	Eigenvector: 3.3217313E-4	Information: 4.3198524	LAC: 5.0	Betweenness: 18.480215	Closeness: 0.27476636	Network: 5.885714
MRVI1	Subgragh: 106.92905	Degree: 2.0	Eigenvector: 4.2298945E-4	Information: 2.2187285	LAC: 1.0	Betweenness: 0.0	Closeness: 0.21366279	Network: 2.0
MAPK3	Subgragh: 3763.8364	Degree: 12.0	Eigenvector: 3.7976808E-4	Information: 5.056548	LAC: 7.1666665	Betweenness: 189.07948	Closeness: 0.2773585	Network: 9.107575
MAML1	Subgragh: 562.3916	Degree: 6.0	Eigenvector: 5.287678E-4	Information: 3.8050835	LAC: 5.0	Betweenness: 0.0	Closeness: 0.2318612	Network: 6.0
LFNG	Subgragh: 562.3916	Degree: 6.0	Eigenvector: 5.287678E-4	Information: 3.8050835	LAC: 5.0	Betweenness: 0.0	Closeness: 0.2318612	Network: 6.0
KRAS	Subgragh: 2572.499	Degree: 10.0	Eigenvector: 1.917015E-4	Information: 4.7267814	LAC: 6.4	Betweenness: 37.26822	Closeness: 0.23863636	Network: 7.698413
KCNQ1	Subgragh: 3949087.0	Degree: 19.0	Eigenvector: 0.09939127	Information: 5.840981	LAC: 6.5263157	Betweenness: 273.42935	Closeness: 0.33485195	Network: 9.969048
KCNJ3	Subgragh: 45258.88	Degree: 11.0	Eigenvector: 0.0032306705	Information: 4.8998713	LAC: 6.5454545	Betweenness: 5123.4834	Closeness: 0.34186047	Network: 7.2
KCNH2	Subgragh: 3661083.0	Degree: 19.0	Eigenvector: 0.09569581	Information: 5.840981	LAC: 8.0	Betweenness: 1569.6685	Closeness: 0.3868421	Network: 13.832635
KCNE4	Subgragh: 872963.94	Degree: 9.0	Eigenvector: 0.046712097	Information: 4.534564	LAC: 6.888889	Betweenness: 1.3160173	Closeness: 0.3094737	Network: 8.0
KCNE3	Subgragh: 1119027.5	Degree: 11.0	Eigenvector: 0.052885454	Information: 4.8998713	LAC: 7.2727275	Betweenness: 7.2209845	Closeness: 0.31477517	Network: 8.7
KCNE2	Subgragh: 1147353.0	Degree: 11.0	Eigenvector: 0.053554904	Information: 4.8998713	LAC: 6.181818	Betweenness: 9.714327	Closeness: 0.31410256	Network: 7.383333
KCNE1L	Subgragh: 507006.88	Degree: 7.0	Eigenvector: 0.035596468	Information: 4.078461	LAC: 5.714286	Betweenness: 0.36363637	Closeness: 0.3081761	Network: 6.6666665
KCNE1	Subgragh: 2214865.5	Degree: 16.0	Eigenvector: 0.07441355	Information: 5.5583854	LAC: 7.125	Betweenness: 383.24084	Closeness: 0.3325792	Network: 9.327381
KCNAB3	Subgragh: 270446.44	Degree: 5.0	Eigenvector: 0.025997372	Information: 3.4929101	LAC: 2.4	Betweenness: 0.6062271	Closeness: 0.28767124	Network: 3.0
KCNAB2	Subgragh: 300071.12	Degree: 6.0	Eigenvector: 0.027381862	Information: 3.8050835	LAC: 3.6666667	Betweenness: 0.6062271	Closeness: 0.2882353	Network: 4.4
KCNAB1	Subgragh: 300071.12	Degree: 6.0	Eigenvector: 0.027381862	Information: 3.8050835	LAC: 3.6666667	Betweenness: 0.6062271	Closeness: 0.2882353	Network: 4.4
KCNA5	Subgragh: 2099823.2	Degree: 9.0	Eigenvector: 0.07249222	Information: 4.534564	LAC: 4.6666665	Betweenness: 8.1409	Closeness: 0.31545064	Network: 5.25
KCNA4	Subgragh: 3117122.5	Degree: 13.0	Eigenvector: 0.08832019	Information: 5.199043	LAC: 5.8461537	Betweenness: 65.50372	Closeness: 0.3181818	Network: 7.4166665
KCNA2	Subgragh: 5533279.5	Degree: 18.0	Eigenvector: 0.11767032	Information: 5.753736	LAC: 8.888889	Betweenness: 91.813446	Closeness: 0.32166302	Network: 12.1784315
KCNA1	Subgragh: 5140888.5	Degree: 17.0	Eigenvector: 0.11342248	Information: 5.6598024	LAC: 8.0	Betweenness: 90.41516	Closeness: 0.3209607	Network: 10.2625
KALRN	Subgragh: 453841.8	Degree: 5.0	Eigenvector: 0.033703856	Information: 3.4929101	LAC: 4.0	Betweenness: 0.0	Closeness: 0.27579737	Network: 5.0
JUP	Subgragh: 4267.0215	Degree: 13.0	Eigenvector: 4.084699E-4	Information: 5.1990433	LAC: 7.3846154	Betweenness: 85.24523	Closeness: 0.27788278	Network: 9.882973
ITPR3	Subgragh: 421.51962	Degree: 1.0	Eigenvector: 0.001016006	Information: 1.6256909	LAC: 0.0	Betweenness: 0.0	Closeness: 0.26775956	Network: 0.0
HRAS1	Subgragh: 3238.9348	Degree: 12.0	Eigenvector: 2.1206803E-4	Information: 5.056548	LAC: 6.5	Betweenness: 70.89182	Closeness: 0.23941368	Network: 8.600361
HN	Subgragh: 31.88464	Degree: 4.0	Eigenvector: 1.8948226E-4	Information: 3.1330576	LAC: 3.0	Betweenness: 0.0	Closeness: 0.24789207	Network: 4.0
HEY1	Subgragh: 833.76184	Degree: 8.0	Eigenvector: 6.1055785E-4	Information: 4.319852	LAC: 6.25	Betweenness: 1.0	Closeness: 0.23259494	Network: 7.714286
GNGT1	Subgragh: 115765.13	Degree: 16.0	Eigenvector: 3.8719235E-4	Information: 5.5583854	LAC: 12.875	Betweenness: 243.41962	Closeness: 0.2658228	Network: 15.646153
GNG8	Subgragh: 115765.13	Degree: 16.0	Eigenvector: 3.8719235E-4	Information: 5.5583854	LAC: 12.875	Betweenness: 243.41962	Closeness: 0.2658228	Network: 15.646153
GNG7	Subgragh: 115765.13	Degree: 16.0	Eigenvector: 3.8719235E-4	Information: 5.5583854	LAC: 12.875	Betweenness: 243.41962	Closeness: 0.2658228	Network: 15.646153
GNG5	Subgragh: 115765.13	Degree: 16.0	Eigenvector: 3.8719235E-4	Information: 5.5583854	LAC: 12.875	Betweenness: 243.41962	Closeness: 0.2658228	Network: 15.646153
GNG3	Subgragh: 115765.13	Degree: 16.0	Eigenvector: 3.8719235E-4	Information: 5.5583854	LAC: 12.875	Betweenness: 243.41962	Closeness: 0.2658228	Network: 15.646153
GNG2	Subgragh: 115765.13	Degree: 16.0	Eigenvector: 3.8719235E-4	Information: 5.5583854	LAC: 12.875	Betweenness: 243.41962	Closeness: 0.2658228	Network: 15.646153
GNB3	Subgragh: 115765.13	Degree: 16.0	Eigenvector: 3.8719235E-4	Information: 5.5583854	LAC: 12.875	Betweenness: 243.41962	Closeness: 0.2658228	Network: 15.646153
GNB1	Subgragh: 87105.516	Degree: 13.0	Eigenvector: 1.9927716E-4	Information: 5.199043	LAC: 12.0	Betweenness: 0.0	Closeness: 0.21491228	Network: 13.0
GNAZ	Subgragh: 88794.266	Degree: 14.0	Eigenvector: 2.0068041E-4	Information: 5.329195	LAC: 11.285714	Betweenness: 34.258373	Closeness: 0.21617647	Network: 12.557693
GNAT2	Subgragh: 87105.52	Degree: 13.0	Eigenvector: 1.9927716E-4	Information: 5.199043	LAC: 12.0	Betweenness: 0.0	Closeness: 0.21491228	Network: 13.0
GNAT1	Subgragh: 87105.52	Degree: 13.0	Eigenvector: 1.9927716E-4	Information: 5.199043	LAC: 12.0	Betweenness: 0.0	Closeness: 0.21491228	Network: 13.0
GNAO1	Subgragh: 88794.266	Degree: 14.0	Eigenvector: 2.0068041E-4	Information: 5.329195	LAC: 11.285714	Betweenness: 34.258373	Closeness: 0.21617647	Network: 12.557693
GNAI2	Subgragh: 106893.1	Degree: 15.0	Eigenvector: 2.3181389E-4	Information: 5.4485474	LAC: 12.666667	Betweenness: 2.6432748	Closeness: 0.21554253	Network: 14.646153
GNAI1	Subgragh: 106893.07	Degree: 15.0	Eigenvector: 2.3181389E-4	Information: 5.4485474	LAC: 12.666667	Betweenness: 2.6432748	Closeness: 0.21554253	Network: 14.646153
GJA1	Subgragh: 8264.481	Degree: 14.0	Eigenvector: 0.0033266202	Information: 5.329195	LAC: 6.428571	Betweenness: 3844.0962	Closeness: 0.35083532	Network: 9.509058
GABBR2	Subgragh: 59275.375	Degree: 11.0	Eigenvector: 3.396352E-4	Information: 4.8998713	LAC: 9.636364	Betweenness: 57.252525	Closeness: 0.26296958	Network: 10.6
GABBR1	Subgragh: 59275.375	Degree: 11.0	Eigenvector: 3.396352E-4	Information: 4.8998713	LAC: 9.636364	Betweenness: 57.252525	Closeness: 0.26296958	Network: 10.6

FKBP1B	Subgragh: 3077325.2	Degree: 18.0	Eigenvector: 0.08773326	Information: 5.753736	LAC: 8.0	Betweenness: 363.59512	Closeness: 0.34186047	Network: 13.009923
FGF12	Subgragh: 1986759.8	Degree: 7.0	Eigenvector: 0.07052335	Information: 4.078461	LAC: 4.285714	Betweenness: 3.893985	Closeness: 0.31144068	Network: 5.0
ENSCPOGO(	Subgragh: 1.0517248E7	Degree: 19.0	Eigenvector: 0.1622583	Information: 5.8409815	LAC: 12.631579	Betweenness: 129.00198	Closeness: 0.3195652	Network: 14.723077
DYSF	Subgragh: 1.9637932	Degree: 1.0	Eigenvector: 2.8053699E-5	Information: 1.6256909	LAC: 0.0	Betweenness: 0.0	Closeness: 0.20792079	Network: 0.0
DTX1	Subgragh: 1114.6998	Degree: 7.0	Eigenvector: 0.0011137608	Information: 4.078461	LAC: 6.0	Betweenness: 0.0	Closeness: 0.25565216	Network: 7.0
DNM2	Subgragh: 2740.5774	Degree: 12.0	Eigenvector: 3.6690632E-4	Information: 5.056548	LAC: 6.1666665	Betweenness: 220.37039	Closeness: 0.2773585	Network: 8.783549
DNM1	Subgragh: 3214.8662	Degree: 13.0	Eigenvector: 3.766095E-4	Information: 5.1990433	LAC: 6.3076925	Betweenness: 252.23557	Closeness: 0.27788278	Network: 9.387085
DHPR	Subgragh: 3993.0642	Degree: 11.0	Eigenvector: 0.0031435103	Information: 4.8998713	LAC: 2.0	Betweenness: 2797.3333	Closeness: 0.32666665	Network: 7.0
CTNNB1	Subgragh: 6844.1094	Degree: 19.0	Eigenvector: 0.0013287772	Information: 5.8409815	LAC: 7.1578946	Betweenness: 1289.7253	Closeness: 0.30309278	Network: 13.742354
CLTC	Subgragh: 648.6563	Degree: 7.0	Eigenvector: 8.8033616E-5	Information: 4.078461	LAC: 4.285714	Betweenness: 4.6666665	Closeness: 0.22138554	Network: 5.0
CLTA	Subgragh: 909.60785	Degree: 8.0	Eigenvector: 2.4727712E-4	Information: 4.3198524	LAC: 5.5	Betweenness: 142.33772	Closeness: 0.26630434	Network: 6.5238094
CG3603	Subgragh: 12.946825	Degree: 1.0	Eigenvector: 1.5892624E-4	Information: 1.6256909	LAC: 0.0	Betweenness: 0.0	Closeness: 0.24664429	Network: 0.0
CG12116	Subgragh: 22.947414	Degree: 3.0	Eigenvector: 1.7890391E-4	Information: 2.7136943	LAC: 2.0	Betweenness: 0.0	Closeness: 0.24747474	Network: 3.0
CDH2	Subgragh: 3406.994	Degree: 11.0	Eigenvector: 3.752259E-4	Information: 4.8998713	LAC: 7.818182	Betweenness: 75.21795	Closeness: 0.27683616	Network: 9.330556
CAV3	Subgragh: 131.21785	Degree: 2.0	Eigenvector: 5.5654056E-4	Information: 2.2187285	LAC: 0.0	Betweenness: 292.0	Closeness: 0.2620321	Network: 0.0
CASQ2	Subgragh: 585340.56	Degree: 8.0	Eigenvector: 0.03825732	Information: 4.3198524	LAC: 5.75	Betweenness: 20.761045	Closeness: 0.3245033	Network: 6.714286
CALM2	Subgragh: 6666662.5	Degree: 16.0	Eigenvector: 0.12917565	Information: 5.5583854	LAC: 10.375	Betweenness: 47.59109	Closeness: 0.33333334	Network: 10.851082
CAMK2D	Subgragh: 1.1205029E7	Degree: 20.0	Eigenvector: 0.16747928	Information: 5.9222302	LAC: 13.9	Betweenness: 23.00488	Closeness: 0.318872	Network: 15.088343
CAMK2B	Subgragh: 1.7749196E7	Degree: 27.0	Eigenvector: 0.21078438	Information: 6.36491	LAC: 14.740741	Betweenness: 133.42503	Closeness: 0.34106728	Network: 19.831997
CAMK2A	Subgragh: 3.1768708E7	Degree: 46.0	Eigenvector: 0.28200343	Information: 6.9994287	LAC: 13.695652	Betweenness: 1833.6877	Closeness: 0.37595907	Network: 36.7912
CALML6	Subgragh: 5286932.5	Degree: 14.0	Eigenvector: 0.11503584	Information: 5.329195	LAC: 9.571428	Betweenness: 17.514671	Closeness: 0.30625	Network: 10.346765
CALML5	Subgragh: 5286932.5	Degree: 14.0	Eigenvector: 0.11503584	Information: 5.329195	LAC: 9.571428	Betweenness: 17.514671	Closeness: 0.30625	Network: 10.346765
CALML3	Subgragh: 5286932.5	Degree: 14.0	Eigenvector: 0.11503584	Information: 5.329195	LAC: 9.571428	Betweenness: 17.514671	Closeness: 0.30625	Network: 10.346765
CALM3	Subgragh: 6553722.5	Degree: 16.0	Eigenvector: 0.1280786	Information: 5.5583854	LAC: 8.5	Betweenness: 40.35117	Closeness: 0.3081761	Network: 9.614862
CAMK2G	Subgragh: 2.7757722E7	Degree: 42.0	Eigenvector: 0.26360258	Information: 6.9050612	LAC: 12.904762	Betweenness: 1983.0173	Closeness: 0.37309644	Network: 30.45621
CALM1	Subgragh: 1.0691622E7	Degree: 23.0	Eigenvector: 0.16358083	Information: 6.1355953	LAC: 12.0	Betweenness: 102.26587	Closeness: 0.33870968	Network: 17.070618
CACNA1S	Subgragh: 100023.67	Degree: 4.0	Eigenvector: 0.015801962	Information: 3.1330576	LAC: 3.0	Betweenness: 0.0	Closeness: 0.3006135	Network: 4.0
CACNA1D	Subgragh: 2245365.5	Degree: 12.0	Eigenvector: 0.074946664	Information: 5.056548	LAC: 8.0	Betweenness: 30.625074	Closeness: 0.32594234	Network: 9.172619
CACNA1C	Subgragh: 2441499.2	Degree: 13.0	Eigenvector: 0.07814964	Information: 5.199043	LAC: 8.461538	Betweenness: 32.11317	Closeness: 0.32666665	Network: 10.076839
CACNA1A	Subgragh: 1782917.5	Degree: 10.0	Eigenvector: 0.06679289	Information: 4.7267814	LAC: 5.4	Betweenness: 19.079866	Closeness: 0.3012295	Network: 6.0277777
BCAR1	Subgragh: 705.79974	Degree: 5.0	Eigenvector: 6.912282E-5	Information: 3.4929106	LAC: 4.0	Betweenness: 0.0	Closeness: 0.21973094	Network: 5.0
ATP2A1	Subgragh: 163617.88	Degree: 12.0	Eigenvector: 0.02019249	Information: 5.056548	LAC: 2.8333333	Betweenness: 2260.6877	Closeness: 0.36476427	Network: 5.190909
ASPH	Subgragh: 420553.8	Degree: 9.0	Eigenvector: 0.03240384	Information: 4.534564	LAC: 5.5555553	Betweenness: 9.890683	Closeness: 0.2987805	Network: 6.607143
AP2S1	Subgragh: 682.4279	Degree: 7.0	Eigenvector: 8.156701E-5	Information: 4.078461	LAC: 4.571429	Betweenness: 3.8675325	Closeness: 0.22171946	Network: 5.3333335
AP2M1	Subgragh: 909.60785	Degree: 8.0	Eigenvector: 2.4727712E-4	Information: 4.3198524	LAC: 5.5	Betweenness: 142.33772	Closeness: 0.26630434	Network: 6.5238094
AP2B1	Subgragh: 912.96704	Degree: 8.0	Eigenvector: 9.0619804E-5	Information: 4.3198524	LAC: 4.25	Betweenness: 12.182684	Closeness: 0.22205438	Network: 5.1190476
AKAP9	Subgragh: 1251024.5	Degree: 13.0	Eigenvector: 0.05590674	Information: 5.1990433	LAC: 6.923077	Betweenness: 262.2608	Closeness: 0.3325792	Network: 8.114285
AKAP6	Subgragh: 244482.52	Degree: 7.0	Eigenvector: 0.02470182	Information: 4.078461	LAC: 5.714286	Betweenness: 0.25	Closeness: 0.2945892	Network: 6.6666665
AKAP1	Subgragh: 65148.277	Degree: 8.0	Eigenvector: 0.012643386	Information: 4.3198524	LAC: 6.5	Betweenness: 1.4888889	Closeness: 0.25925925	Network: 7.428571
ADE3	Subgragh: 12.946826	Degree: 1.0	Eigenvector: 1.5892624E-4	Information: 1.6256909	LAC: 0.0	Betweenness: 0.0	Closeness: 0.24664429	Network: 0.0
ADCY8	Subgragh: 43295.93	Degree: 9.0	Eigenvector: 0.010243264	Information: 4.534564	LAC: 6.6666665	Betweenness: 36.298462	Closeness: 0.25925925	Network: 7.5
ACLY	Subgragh: 26.816294	Degree: 3.0	Eigenvector: 1.03786915E-5	Information: 2.7136943	LAC: 2.0	Betweenness: 0.0	Closeness: 0.20851064	Network: 3.0
