# Supplementary material for: A Comprehensive In Silico Method to Study the QSTR of the Aconitine Alkaloids for Designing Novel Drugs
Source: Molecules. 2018 Sep 18;23(9):2385. doi: 10.3390/molecules23092385 (PMC6225272; doi:10.3390/molecules23092385)
Supplement: Supplementary file 1 [file molecules-23-02385-s001.zip › molecules-354597-supplementary/Table S2.pdf]

Table S2. The dock result of the Aconitine alkaloids to the protein 2V70 and 2VZ6.

ligand	receptor	rseq	mseq	S	rmsd_refi	FP:PLIF	PLIF_ligidx	E_conf	E_place	E_score1	E_refine	E_score2
1	2V70	1	1	-9.03862	2.427852	10	[[4,11,26,40,47,48,58,60,90,91]]	227.2475	-96.5353	-7.34918	-37.8371	-9.03862
1	2V70	1	1	-8.84172	2.149052	10	[[4,26,28,40,47,48,58,60,90,91]]	235.1082	-91.3857	-6.34467	-39.3511	-8.84172
1	2V70	1	1	-8.54948	2.044616	29 32	[9,9]	251.7127	-51.1315	-6.93746	-37.4023	-8.54948
1	2V70	1	1	-8.35709	3.198298		[]	227.0408	-81.6554	-6.55824	-33.1959	-8.35709
1	2V70	1	1	-7.95816	2.816367	10	[[1,6,13,29,42,49,55,61,86,96]]	260.9932	-85.0802	-6.6492	-25.6133	-7.95816
2	2V70	1	2	-7.06671	1.777539		[]	79.98949	-81.3195	-8.28233	-29.8395	-7.06671
2	2V70	1	2	-6.99717	3.432195		[]	82.13496	-90.6506	-7.70791	-30.4801	-6.99717
2	2V70	1	2	-6.93976	1.985647	2 3 6 7	[35,35,10,10]	77.88571	-92.5891	-8.8053	-35.9428	-6.93976
2	2V70	1	2	-6.67518	1.70978	9 23	[35,35]	75.39453	-89.7905	-8.51903	-32.0996	-6.67518
2	2V70	1	2	-6.63468	2.031292		[]	98.23914	-95.4309	-7.72429	-25.6541	-6.63468
3	2V70	1	3	-8.70672	2.243983	6 10	[27,[4,5,12,19,21,22,44,70,71,73]]	494.3481	-56.1207	-8.13103	-46.4695	-8.70672
3	2V70	1	3	-8.05286	1.90219	8 10	[[10,19,12,22,21,11],[5,12,21,22,44,73,74,88,11,19]]	492.4698	-85.2657	-8.12601	-46.1814	-8.05286
3	2V70	1	3	-7.89897	1.700932	10	[[4,10,11,19,21,43,69,86,87,12]]	492.0929	-100.38	-8.28189	-39.0053	-7.89897
3	2V70	1	3	-7.87525	1.87171	4 5	[41,41]	466.7637	-75.9928	-8.66042	-45.0922	-7.87525
3	2V70	1	3	-7.87323	2.187611		[]	486.8844	-64.5875	-7.85279	-40.9596	-7.87323
4	2V70	1	4	-7.3243	1.692895	10	[[2,6,7,14,16,22,28,31,34,38]]	758.7833	-86.7292	-8.22968	-25.7939	-7.3243
4	2V70	1	4	-6.92389	1.296806		[]	749.9845	-99.7506	-8.97979	-30.2124	-6.92389
4	2V70	1	4	-6.83269	1.641392	10	[[8,17,23,24,30,33,56,57,58,67]]	753.2866	-85.2242	-8.24302	-27.5488	-6.83269
4	2V70	1	4	-6.74276	2.185868	10	[[8,17,23,24,30,33,56,57,58,67]]	749.7653	-80.5765	-9.42271	-27.6563	-6.74276
4	2V70	1	4	-6.71816	2.597223	2 3	[22,22]	768.7506	-98.2124	-8.92557	-19.9417	-6.71816
5	2V70	1	5	-8.55681	2.279317		[]	156.9359	-96.4442	-8.43496	-29.348	-8.55681
5	2V70	1	5	-7.99676	1.698724	9 10	[24,[2,6,7,15,16,17,24,31,46,47]]	169.0559	-75.719	-8.40465	-33.1127	-7.99676

5	2V70	1	5	-7.89047	3.210677					[]	176.2225	-115.269	-9.42816	-30.3467	-7.89047
5	2V70	1	5	-7.87579	2.431977					[]	152.9026	-88.6273	-8.5056	-36.3366	-7.87579
5	2V70	1	5	-7.69998	1.396457					[]	150.7074	-95.7147	-8.76545	-21.5775	-7.69998
5	2V70	1	5	-7.59413	1.447709					[]	148.8331	-97.4109	-9.94235	-20.3189	-7.59413
5	2V70	1	5	-7.58224	2.157373	2	3	16	17	[39,39,[38,42,44,45,43,41],[38,42,44,45,43,41]]	135.1118	-75.3251	-8.23534	-42.8821	-7.58224
5	2V70	1	5	-7.28864	1.541547				9	30	163.9472	-103.25	-10.2749	-20.5326	-7.28864
5	2V70	1	5	-7.07434	1.3035					[]	146.0965	-107.233	-8.63068	-26.934	-7.07434
5	2V70	1	5	-6.91388	2.795189					[]	149.4369	-88.2537	-8.44082	-26.8545	-6.91388
6	2V70	1	6	-8.97547	1.607837				10	[[3,6,8,11,17,19,21,25,30,34]]	171.9381	-94.2011	-9.03963	-35.2364	-8.97547
6	2V70	1	6	-8.07966	1.976699	20	21			[[42,47,46,45,44,43],[42,47,46,45,44,43]]	191.2277	-74.6032	-8.31176	-30.3711	-8.07966
6	2V70	1	6	-7.86863	2.677291					[]	186.0519	-113.068	-8.18683	-33.7544	-7.86863
6	2V70	1	6	-7.53892	1.143091					[]	159.8065	-91.9628	-8.15141	-35.1847	-7.53892
6	2V70	1	6	-7.53091	1.967047				9	41	152.4483	-82.1281	-8.38087	-35.4636	-7.53091
7	2V70	1	7	-8.04035	1.890098				24	[[79,81,9,11,8,30,39,18,40,56]]	559.524	-101.218	-7.95172	-17.7824	-8.04035
7	2V70	1	7	-7.94456	1.528403	25	26		11	[37,37,40,[11,18,30,39,40,56,81,93,9,45]]	529.8604	-91.9587	-9.20801	-32.0065	-7.94456
7	2V70	1	7	-7.44615	1.568086	4	5	10		[40,40,[11,39,81,9,18,30,79,4,42,56]]	519.4249	-86.0193	-7.92431	-38.3406	-7.44615
7	2V70	1	7	-7.18605	1.520319	18	25		11	[43,37,40,40,[8,9,10,11,12,18,28,30,40,45],[8,10,12,18,11,9]]	602.0568	-117.606	-8.44204	-28.0799	-7.18605
7	2V70	1	7	-7.17983	1.735773	2	3	6	16	[38,38,38,[8,10,12,18,11,9],[8,10,12,18,11,9]]	532.119	-81.6785	-8.81142	-37.762	-7.17983
8	2V70	1	8	-8.4111	1.821191	15	41			[38,38]	328.8668	-81.3038	-8.31616	-37.6118	-8.4111
8	2V70	1	8	-8.19024	2.822495	9	23			[41,41]	445.2945	-99.2971	-8.3298	-28.0126	-8.19024
8	2V70	1	8	-7.61978	2.550316				22	[[16,16,16,16,16,16]]	504.6905	-104.086	-9.13315	-21.3824	-7.61978
8	2V70	1	8	-7.30536	1.769909				15	36	455.5769	-80.6875	-8.48862	-37.9832	-7.30536

8	2V70	1	8	-7.29592	1.620976	29	20	6	7	[38,[7,9,11,18,10,8],36,36,[23,41,48,49,60,84,86,2,5,15]]		462.3796	-72.8994	-9.0467	-30.5946	-7.29592
9	2V70	1	9	-8.08291	1.506813	10	16			[[9,10,11,12,13,21,82,83,85,92],[9,11,13,21,12,10]]		473.2804	-74.0305	-7.9291	-32.1872	-8.08291
9	2V70	1	9	-8.01173	3.20686					[]		452.8512	-78.8528	-8.55623	-37.1598	-8.01173
9	2V70	1	9	-7.87342	1.683797	37	38	11		[43,43,41,41,[9,10,11,12,13,21,32,41,47,82]]		483.3424	-77.6858	-9.06076	-44.6757	-7.87342
9	2V70	1	9	-7.77818	1.286089					[]		471.2479	-59.3281	-8.45595	-31.2031	-7.77818
9	2V70	1	9	-7.52555	2.214701					[]		480.3321	-77.5314	-7.92495	-31.008	-7.52555
10	2V70	1	10	-7.53951	2.115426			35			11	363.5805	-72.032	-8.87387	-39.9745	-7.53951
10	2V70	1	10	-7.30426	3.104195	35	10			[11,[9,27,59,62,64,7,19,29,30,37]]		354.5447	-83.8742	-9.2447	-36.6144	-7.30426
10	2V70	1	10	-7.27337	2.372484					[]		402.1701	-90.6637	-8.95859	-33.8326	-7.27337
10	2V70	1	10	-7.16903	4.104233	6	7			[6,6]		371.8395	-84.1	-10.128	-31.9548	-7.16903
10	2V70	1	10	-7.02567	2.850243	6	10			[31,[6,26,49,51,61,79,1,86,53,2]]		362.5105	-86.0778	-9.05418	-29.5092	-7.02567
12	2V70	1	12	-8.83127	2.545199			13		[[30,32,34,35,33,31]]		529.9581	-104.77	-9.56623	-44.6852	-8.83127
12	2V70	1	12	-8.47186	3.140958	22	31	6	1	[[27,27,27,27,27,27],[27,27,27,27,27,27],25,[5,26,27,28,45,70,71,72,92,4]]		951.5167	-98.0523	-10.3129	-42.504	-8.47186
12	2V70	1	12	-8.37121	4.020075			29			48	330.2833	-70.6992	-8.71804	-48.8549	-8.37121
12	2V70	1	12	-8.30504	3.428274	29	32	22		[6,6,[27,27,27,27,27,27]]		432.0944	-101.211	-8.39253	-48.1546	-8.30504
12	2V70	1	12	-8.18209	5.11342	6	10			[25,[5,30,31,32,33,34,35,75,76,4]]		951.0066	-78.667	-8.40753	-46.5879	-8.18209
13	2V70	1	13	-8.27453	1.972244			9			24	104.6532	-73.2689	-8.96562	-47.0989	-8.27453
13	2V70	1	13	-7.98081	2.012662	2	3			[39,39]		124.7611	-80.523	-8.25308	-33.1728	-7.98081
13	2V70	1	13	-7.69105	1.771878	33	34			[[42,46,48,49,47,45],[42,46,48,49,47,45]]		107.994	-67.2324	-8.50867	-46.0397	-7.69105
13	2V70	1	13	-7.67838	1.453738					[]		107.4603	-103.903	-8.80725	-43.2507	-7.67838

13	2V70	1	13	-7.56053	2.581646	1	[[42,46,48,49,47,45,42,46,48,49,47,45]]	90.42992	-101.34	-10.0859	-42.683	-7.56053
14	2V70	1	14	-8.50253	2.021634	10 27 28	[[27,28,31,34,37,38,39,40,42,43],46,46]	172.1957	-96.3716	-9.81493	-28.1329	-8.50253
14	2V70	1	14	-8.22888	1.984356	29 30 6 7	[13,[34,38,41,44,40,37],15,15,[35,43,45,47,49,96,97,98,99,31]]	161.7405	-79.6915	-9.11426	-47.5047	-8.22888
14	2V70	1	14	-8.16614	1.718532		[]	151.9247	-65.8207	-9.226	-41.5291	-8.16614
14	2V70	1	14	-8.04381	2.373407	9 23	[33,33]	162.3895	-81.1548	-9.4899	-30.3152	-8.04381
14	2V70	1	14	-7.86271	1.472629		[]	165.9002	-99.0918	-9.21577	-31.58	-7.86271
15	2V70	1	15	-8.41091	2.876873		[]	433.0884	-69.3985	-8.10522	-36.6378	-8.41091
15	2V70	1	15	-7.91762	2.267237		[]	427.4212	-69.5576	-7.10369	-28.6541	-7.91762
15	2V70	1	15	-7.4637	1.556922	29 2 10	[45,40,[66,83,2,8,65,84,85,20,67,18]]	404.7519	-65.325	-7.8716	-39.3662	-7.4637
15	2V70	1	15	-7.45385	2.351712		[]	329.5383	-44.5559	-7.09636	-35.7961	-7.45385
15	2V70	1	15	-7.4209	1.765784	29 32 2 3	[42,42,41,41]	457.5509	-103.435	-9.16302	-34.8332	-7.4209
16	2V70	1	16	-8.82691	1.299715	10	[[8,19,28,33,35,36,38,39,41,44]]	186.1039	-64.1526	-8.63153	-31.8731	-8.82691
16	2V70	1	16	-7.48055	2.47845	1	[[35,39,41,42,40,38]]	186.6794	-84.6528	-8.96437	-33.5396	-7.48055
16	2V70	1	16	-7.40497	1.997007	10	[[25,38,40,42,67,68,69,81,83,85]]	178.8833	-95.6132	-9.19327	-27.2052	-7.40497
16	2V70	1	16	-7.39196	2.61204		[]	191.1956	-114.432	-8.69949	-26.9069	-7.39196
16	2V70	1	16	-7.1443	3.35688		[]	161.0556	-65.7955	-8.63205	-40.9027	-7.1443
17	2V70	1	17	-7.87267	2.088966	4 5 10	[28,28,[3,9,13,18,20,29,48,57,60,63]]	200.8252	-132.348	-9.80888	-28.9656	-7.87267
17	2V70	1	17	-7.7856	2.282742	9	21	214.8013	-82.6065	-8.32556	-18.3469	-7.7856
17	2V70	1	17	-7.61849	2.41147		[]	152.127	-64.0278	-8.38861	-36.5608	-7.61849
17	2V70	1	17	-7.34475	1.699139	1	[[40,44,46,47,45,43]]	162.6613	-68.9205	-10.1884	-36.4417	-7.34475
17	2V70	1	17	-7.32761	2.471305	11 9 23	[16,30,30]	224.9754	-57.4959	-8.26337	-22.1522	-7.32761
18	2V70	1	18	-7.84931	2.04398	10	[[34,38,83,37,85,92,70,27,84,10]]	117.4876	-73.2558	-8.58161	-37.451	-7.84931
18	2V70	1	18	-7.66074	1.311189		[]	123.6084	-88.6489	-9.74787	-36.1444	-7.66074
18	2V70	1	18	-7.57153	1.393964		[]	264.4735	-88.8796	-8.70142	-33.8187	-7.57153
18	2V70	1	18	-7.36085	1.487717		[]	119.0508	-77.2857	-8.26327	-36.8878	-7.36085

18	2V70	1	18	-7.33275	1.338224	9 23 10	[17,17,[2,7,10,17,25,27,34,47,49,53]]	142.435	-85.0244	-8.90031	-3.06561	-7.33275
19	2V70	1	19	-7.53199	2.255959		[]	290.0166	-75.3791	-6.91524	-28.098	-7.53199
19	2V70	1	19	-7.14192	1.653783		[]	326.5758	-85.9072	-6.85281	-15.2005	-7.14192
19	2V70	1	19	-7.10606	2.543881		[]	278.2257	-66.9209	-6.96973	-30.4902	-7.10606
19	2V70	1	19	-7.05676	1.969371	9	35	278.9125	-62.073	-6.97259	-26.5885	-7.05676
19	2V70	1	19	-7.04228	2.205437	37 38	[37,37]	340.7403	-71.5247	-6.91109	-20.2113	-7.04228
20	2V70	1	20	-9.36105	3.345224	36 37 38	[49,13,13,28]	674.705	-58.7291	-6.13741	-53.64	-9.36105
20	2V70	1	20	-9.32166	3.831885	2 37 38	[12,13,13]	701.945	-94.5879	-6.98949	-50.6011	-9.32166
20	2V70	1	20	-9.07452	4.110587		[]	853.6174	-64.6296	-6.4024	-38.7506	-9.07452
20	2V70	1	20	-8.59912	4.261309	6 10 16 1	[28,[48,51,52,53,54,55,79,106,108,37],[48,52,54,55,53,51],[48,52,54,55,53,51]]	686.2805	-78.8413	-6.67116	-50.2244	-8.59912
20	2V70	1	20	-8.1529	3.415794		[]	718.2269	-66.9044	-7.1361	-41.2041	-8.1529
21	2V70	1	21	-8.09683	1.936526	10	[[4,5,23,24,25,35,36,40,41,42]]	417.3049	-119.652	-9.21589	-22.6361	-8.09683
21	2V70	1	21	-7.80872	2.41855	6	7	390.1728	-107.439	-8.69154	-26.5378	-7.80872
21	2V70	1	21	-7.60004	2.09678	6	7	419.142	-123.855	-9.60748	-17.465	-7.60004
21	2V70	1	21	-7.49605	2.635899	6 35 10	[32,12,[1,6,34,39,52,80,85,86,27,37]]	378.177	-82.2361	-8.71449	-30.3806	-7.49605
21	2V70	1	21	-7.20202	3.76473	4	6	427.7049	-103.598	-9.02354	-26.8551	-7.20202
22	2V70	1	22	-7.5543	1.879724	11 12 13	[31,31,[36,40,42,43,41,39],[36,40,42,43,41,39]]	157.916	-85.8462	-8.62595	-41.725	-7.5543
22	2V70	1	22	-7.47083	2.46857		[]	161.2137	-74.5836	-8.861	-39.9962	-7.47083
22	2V70	1	22	-7.31678	1.863237	15	31	165.3657	-93.8205	-10.3392	-33.9014	-7.31678
22	2V70	1	22	-7.2998	3.259812		[]	167.7164	-90.4961	-8.60243	-33.6036	-7.2998
22	2V70	1	22	-7.25058	2.186588		[]	161.2885	-67.4249	-8.65677	-36.7263	-7.25058
23	2V70	1	23	-7.28681	1.79133		[]	173.178	-100.924	-9.39788	-25.468	-7.28681
23	2V70	1	23	-6.60607	1.881178	6 7	[26,26]	172.5198	-92.996	-9.22994	-24.0445	-6.60607
23	2V70	1	23	-6.46465	1.950306		[]	139.1928	-61.7427	-9.64448	-28.8544	-6.46465
23	2V70	1	23	-6.41296	1.943059	9	23	140.7766	-69.8243	-8.2953	-26.6875	-6.41296
23	2V70	1	23	-6.40917	2.204575	9	23	144.4051	-96.1699	-8.49291	-24.731	-6.40917

24	2V70	1	24	-7.87825	1.352697	22 6 7 10	[[2,2,2,2,2,2],28,28,[2,3,11,20,26,31,43,44,48,49]]	528.2738	-108.522	-8.38824	-29.036	-7.87825
24	2V70	1	24	-7.68251	1.346903	10	[[4,8,12,16,33,35,37,39,54,56]]	461.7964	-93.5163	-9.39529	-33.7147	-7.68251
24	2V70	1	24	-7.67551	1.887196	6 7	[28,28]	376.4544	-92.3264	-8.47307	-27.9128	-7.67551
24	2V70	1	24	-7.597	1.5024	22 6 7 9	[[2,2,2,2,2,2],28,28,31,31,[2,3,6,11,26,31,32,43,49,50]]	492.3109	-115.292	-8.30506	-34.0245	-7.597
24	2V70	1	24	-7.41165	1.997274	4	27	523.444	-88.1692	-8.93672	-32.3782	-7.41165
25	2V70	1	25	-8.4762	2.41928	9	39	362.3969	-105.397	-9.7491	-35.9079	-8.4762
25	2V70	1	25	-8.38915	2.342752	22 9 23 1	[[4,4,4,4,4,4],39,39,[2,4,21,36,37,39,47,49,56,57]]	366.876	-83.5209	-9.85122	-33.6569	-8.38915
25	2V70	1	25	-8.14493	3.547757		[]	348.2345	-97.3618	-9.15724	-36.7215	-8.14493
25	2V70	1	25	-7.28433	2.313627	9 23 10	[40,40,[5,24,26,36,40,44,49,64,65,66]]	460.9652	-100.34	-8.83983	-37.8817	-7.28433
25	2V70	1	25	-7.25844	2.53165	42	33	456.9665	-75.5017	-9.38477	-19.7198	-7.25844
26	2V70	1	26	-8.16168	1.601083	2 3 22 6	[1,1,[23,23,23,23,23,23],1,7,7]	285.1332	-83.5387	-9.50752	-44.3075	-8.16168
26	2V70	1	26	-8.11193	2.018285	6 10	[4,[12,28,39,42,45,47,56,65,66,67]]	285.2218	-66.536	-9.02067	-40.0438	-8.11193
26	2V70	1	26	-7.83158	1.885825	9 23	[7,7]	285.6354	-96.779	-9.78302	-34.3339	-7.83158
26	2V70	1	26	-7.4588	1.329001	13	[[31,33,35,36,34,32]]	269.6613	-96.5349	-8.71861	-37.3164	-7.4588
26	2V70	1	26	-7.45303	1.90269	2 9 23	[1,7,7]	294.1816	-92.7221	-8.70987	-33.522	-7.45303
27	2V70	1	27	-7.63728	1.824114	11 12 10	[39,39,[8,9,11,12,19,30,38,39,44,77],[8,10,12,19,11,9]]	506.6597	-86.628	-9.85615	-34.9635	-7.63728
27	2V70	1	27	-7.60946	1.964618	18 19	[41,39]	507.6846	-79.0664	-8.49174	-30.9126	-7.60946
27	2V70	1	27	-7.55008	2.281815	8 10	[[8,10,12,19,11,9],[8,10,12,78,80,86,9,19,49,76]]	496.1932	-91.0037	-8.91902	-30.359	-7.55008
27	2V70	1	27	-7.34831	2.24599		[]	492.7976	-80.0728	-8.04251	-40.2613	-7.34831
27	2V70	1	27	-7.31709	1.759371		[]	599.5636	-87.931	-8.39848	-14.7834	-7.31709
28	2V70	1	28	-7.62055	2.77948		[]	417.9757	-73.2236	-8.88526	-32.3992	-7.62055

28	2V70	1	28	-7.45612	2.640953			[]	393.3613	-74.0982	-11.164	-41.9267	-7.45612
28	2V70	1	28	-7.34881	3.165079	39 6 7 35		[[37,39,41,42,40,38],25,25,11]	407.6935	-80.0501	-9.11112	-35.9856	-7.34881
28	2V70	1	28	-7.25216	2.755866	6 7 35 40		[11,11,11,11,[10,48,1,82,11,46,49,67,31,69]]	464.6458	-61.3862	-9.20859	-41.9591	-7.25216
28	2V70	1	28	-7.19489	3.359174			[]	401.1649	-112.11	-9.92446	-23.9996	-7.19489
29	2V70	1	29	-7.01274	3.095417			[]	870.8715	-65.2092	-8.07366	-26.7243	-7.01274
29	2V70	1	29	-6.58005	2.580891		22	[[21,21,21,21,21,21]]	900.8123	-89.9526	-7.8767	-25.197	-6.58005
29	2V70	1	29	-6.49496	2.539917	15 10		[5,[3,15,23,32,40,50,51,72,4,6]]	766.6934	-81.1019	-7.87832	-23.9722	-6.49496
29	2V70	1	29	-6.49337	2.848133	11 12 35		[5,5,8]	867.1283	-82.3335	-8.12319	-33.8606	-6.49337
29	2V70	1	29	-6.40405	4.065658	4 6 7		[8,1,1]	880.1988	-83.6712	-10.8312	-31.2456	-6.40405
30	2V70	1	30	-7.04179	3.984906			[]	65.20968	-95.0879	-7.91826	-32.5544	-7.04179
30	2V70	1	30	-7.00929	4.422154			[]	261.4944	-93.4205	-7.98702	-30.2169	-7.00929
30	2V70	1	30	-6.90266	2.976799		10	[[5,10,12,19,20,23,24,26,31,43]]	410.1739	-94.6648	-7.75627	-32.6149	-6.90266
30	2V70	1	30	-6.31044	5.807513	4 5		[25,25]	302.2524	-80.9716	-7.67299	-29.9941	-6.31044
30	2V70	1	30	-6.27117	2.162696		22	[[9,9,9,9,9,9]]	283.3929	-130.722	-8.25986	-11.9615	-6.27117
31	2V70	1	31	-6.59107	1.581163			[]	63.02015	-89.4235	-8.76829	-22.7467	-6.59107
31	2V70	1	31	-6.51837	1.456006	22 9 23		[[15,15,15,15,15,15],7,7]	735.5214	-84.1933	-9.28011	-22.2496	-6.51837
31	2V70	1	31	-6.4966	1.497958		22	[[15,15,15,15,15,15]]	613.8854	-93.025	-8.61483	-17.8279	-6.4966
31	2V70	1	31	-6.48407	2.805569		9	26	612.3973	-79.6527	-9.6271	-28.1351	-6.48407
31	2V70	1	31	-6.48377	0.783044			[]	66.12716	-78.8862	-8.92434	-19.7385	-6.48377
32	2V70	1	32	-8.01096	2.80135			[]	293.3796	-92.9687	-7.92481	-25.0227	-8.01096
32	2V70	1	32	-7.80899	2.344469	1 2 3 4 5		[[39,43,45,46,44,42],24,24,33,33,23,23]	272.1649	-93.4582	-8.388	-43.9536	-7.80899
32	2V70	1	32	-7.60953	1.267651		8	[[39,43,45,46,44,42]]	266.7696	-71.479	-8.54066	-38.4015	-7.60953
32	2V70	1	32	-7.53887	3.044075		9	24	312.1551	-87.1341	-7.83433	-22.705	-7.53887
32	2V70	1	32	-7.30371	1.670573			[]	422.6689	-79.4905	-8.56185	-32.7575	-7.30371
33	2V70	1	33	-8.24803	0.991289			[]	209.7697	-116.431	-10.1276	-32.4018	-8.24803
33	2V70	1	33	-7.97373	0.960303			[]	197.2893	-112.809	-9.5933	-22.6582	-7.97373
33	2V70	1	33	-7.52023	1.341218	6 7		[27,27]	186.9686	-117.117	-8.78118	-39.6242	-7.52023

33	2V70	1	33	-7.44098	1.181069	11 12 10	[27,27,[25,31,34,35,37,38,39,40,41,55],[34,38,40,41,39,37],[34,38,40,41,39,37]]	195.6474	-81.7655	-8.6841	-40.6462	-7.44098
33	2V70	1	33	-7.31469	1.911271	42 43	[30,30]	203.1644	-125.936	-8.87862	-28.7881	-7.31469
1	2VZ6	1	1	-8.06138	1.736709	2 3 19 33	[[53,59,2,7,16,41,48,50,1,5],[6,7,16,41,48,50,51,53,59,60],[3,6,9,10,20,21,48,51,55,59],21,[3,10,21,25,31,34,38,48,51,55],[26,35,40,41,44,59,91,16,43,45],[11,15,39,49,56,66,70,71,85,86],[87,27,28,36,42,71,72,88,89,17],[40,44,46,47,45,43],[32,77,78,79,62,19,58,12,13,22]]	410.9614	-84.3669	-7.66763	-22.216	-8.06138
1	2VZ6	1	1	-7.01644	3.664124	1 2 3 19	[16,[41,16,26,35,53,59,72,90,6,7],[16,26,35,41,51,53,59,90,6,17],[6,10,15,16,21,25,26,34,48,51],[10,21,25,31,34,38,39,55,74,75],[88,36,42,70,80,85,91,24,27,33],[40,44,46,47,45,43],[66,77,12,22,32,49,58,1,4,5]]	184.2367	-87.7497	-5.8029	-21.8844	-7.01644

1	2VZ6	1	1	-6.70149	2.676928	1 2 3 17	[16,[41,59,16,26,28,35,53,90,6,7],[16,26,35,41,51,53,59,2,6,7],38,[3,6,10,16,21,34,38,48,51,55],[10,21,25,31,34,38,39,55,63,74],33,33,[87,24,36,42,70,80,86,89,91,27],[87,88,40,43,45,47,90,92,35,44],[40,44,46,47,45,43],[22,65,66,12,49,58,4,14,19,32]]	191.0564	-58.9116	-5.35072	-21.6298	-6.70149
1	2VZ6	1	1	-6.55493	3.264217	2 3 13 1	[[86,88,89,39,42,70,84,24,34,36,86,88,89,39,42,70,84,24,34,36],[36,42,43,45,88,89,40,90,92,35],[31,55,75,76,10,21,25,38,51,81],[82,30,37,60,83,3,18,20,48,50],[45,46,47,93,94,44,91,43,92,40],21,[52,53,7,17,28,72,59,71,41,16]]	160.9017	-29.3267	-6.27644	-34.0278	-6.55493
1	2VZ6	1	1	-6.30545	1.832358	2 12 13 1	[[37,81,82,38,51,55,75,3,6,10,37,81,82,38,51,55,75,3,6,10],[93,44,46,87,89,91,42,47,94,36],[24,33,39,70,84,85,89,15,25,34],[65,79,78,22,32,66,12,49,56,77],[66,19,22,32,61,62,77,79,12,58]]	182.009	-52.4406	-7.04434	-30.4855	-6.30545

2	2VZ6	1	2	-6.55054	2.356541	2	3	17	18	[[52,3,32,44,70,71,51,53,73,49],[28,43,44,70,73,20,21,32,60,3],28,28,[6,9,16,17,18,20,24,26,28,30],[45,60,70,2,6,11,26,31,47,49],[35,64,5,8,12,34,37,39,42,58],[62,7,13,27,36,37,48,53,63,72],[27,36,68,72,1,5,10,13,22,41]]	397.2436	-48.3138	-6.43338	-18.5647	-6.55054
2	2VZ6	1	2	-6.25361	2.08842	2	3	17	18	[[48,2,31,50,3,11,32,52,53,70],[2,11,31,32,48,52,70,71,3,14],28,28,28,28,[6,9,16,17,20,21,24,26,28,30],27,27,[35,12,34,37,39,42,58,62,64,72],[7,13,27,36,62,63,72,49,50,2],[68,59,1,5,6,10,41,45,47,61]]	408.5316	-82.2021	-7.40477	-16.3366	-6.25361
2	2VZ6	1	2	-6.07435	2.517906	3	19	4	7	[[2,48,49,60,6,11,26,31,61,69],[2,6,11,20,26,43,48,60,61,71],[16,20,28,39,43,61,67,6,17,21],[46,47,1,57,40,66,61,5,9,17],[5,34,58,59,27,42,62,72,7,13]]	352.6574	-41.9854	-6.3533	-27.9233	-6.07435

2	2VZ6	1	2	-5.94692	2.349864	2	3	19	4	[[9,28,38,39,67,73,15,16,21,24],[28,39,67,73,9,16,20,21,24,43],[6,11,16,20,32,39,43,60,61,71],[3,7,11,14,25,31,32,43,48,51],[64,8,29,35,37,51,65,74,12,23],[5,57,58,59,41,18,34,68,17,30]]	370.9131	-33.0788	-7.1487	-16.6245	-5.94692
2	2VZ6	1	2	-5.70135	4.032457	3	17	18	3,	[[5,12,34,35,39,59,64,75,8,16],33,33,33,29,29,[29,74,54,4,56,23,33,38,55,73],[45,46,60,6,28,43,73,1,61,2,45,46,60,6,28,43,73,1,61,2]]	381.8925	-91.9469	-7.59277	-33.2789	-5.70135
3	2VZ6	1	3	-7.35401	1.449889	2	12	13	1	[[49,8,47,53,70,85,2,3,7,18,49,8,47,53,70,85,2,3,7,18],[92,38,45,84,34,37,43,46,83,90],[33,34,38,50,65,72,82,83,37,10],[77,21,31,58,64,76,65,78,11,14],[33,41,82,86,87,88,73,77,65,26],23,23,[23,69,15,60,61,81,6,12,16,56]]	111.9676	-69.8471	-6.99702	-42.0869	-7.35401

3	2VZ6	1	3	-7.30725	1.808435	2	12	40	1[illegible]	[[49,8,47,48,53,63,70,83,3,7,49,8,47,48,53,63,70,83,3,7],[92,45,37,43,46,73,84,85,90,93],38,[33,34,38,50,76,82,84,10,17,18],[77,78,21,31,58,65,64,76,11,14],23,23,[23,69,15,60,62,81,6,12,16,24]]	123.0262	-41.0479	-6.68921	-40.3964	-7.30725
3	2VZ6	1	3	-7.15386	2.648959	2	3	17	18	[[28,35,40,49,63,3,8,19,47,48],[8,19,27,28,47,48,49,53,63,79],17,17,[85,43,45,84,90,92,28,35,39,42],[33,82,26,29,73,74,86,51,54,65],[75,30,36,41,74,87,88,35,39,40],[31,76,77,78,23,58,69,11,14,21]]	172.6986	-97.8612	-6.47124	-21.264	-7.15386
3	2VZ6	1	3	-7.0656	3.887769	2	3	17	18	[[44,39,42,43,45,46,73,89,91,92],[73,88,26,29,33,36,41,74,82,86],36,36,[40,75,30,35,39,63,89,42,87,55],[47,2,7,17,49,53,62,72,1,3]]	130.3706	-68.6514	-6.04032	-36.9055	-7.0656

3	2VZ6	1	3	-7.06488	3.197079	2 3 5 7 8	[[28,35,40,49,63,3,8,19,47,48],[8,19,27,28,47,48,49,53,63,2],[85,43,45,90,92,28,35,39,84,42],[33,82,26,29,73,74,86,51,54,36],[75,30,36,41,74,87,88,35,39,40],[76,31,58,69,11,14,21,59,65,77]]	169.6889	-104.886	-5.97101	-22.3178	-7.06488
4	2VZ6	1	4	-5.38372	3.758449	1 41 3 33	[8,8,[5,8,18,28,29,37,41,43,46,60],2,2,[32,56,68,70,6,26,31,38,42,67,32,56,68,70,6,26,31,38,42,67]]	589.7092	-38.0891	-5.73262	-24.3531	-5.38372
4	2VZ6	1	4	-5.31129	5.88515	28 3 7 11	[[33,74,75,7,58,73,52,53,24,54],[27,45,52,58,75,6,19,42,44,69],[70,71,32,72,56,6,23,26,50,51],[3,39,40,65,1,12,15,59,2,10]]	571.9725	-62.9826	-6.87318	-26.0219	-5.31129
4	2VZ6	1	4	-5.29156	5.133081	28 2 11	[[68,31,4,33,57,67,69,73,75,7],[4,31,57,68,73,27,33,34,47,7],[30,65,66,2,36,49,64,5,41,13]]	456.9686	-80.8419	-6.66209	-28.0195	-5.29156
4	2VZ6	1	4	-5.18139	4.230043	1 41 2 3	[8,8,[5,41,18,36,37,43,2,13,8,29],[5,8,37,41,43,60,61,21,28,29],[23,40,50,51,71,32,70,72,3,1],[33,57,73,74,75,7,27,58,68,69]]	396.6478	-40.446	-5.75154	-26.7138	-5.18139

4	2VZ6	1	4	-5.0708	3.121007	2 3 17 18	[[4,31,67,68,16,23,26,42,50,56],[4,12,15,16,23,31,34,42,50,67],3,3,[60,58,4,21,27,31,34,35,47,57],[36,41,44,48,2,5,13,14,19,22],[7,45,52,55,24,25,54,27,33,69],8,[24,25,46,52,53,54,57,43,61,5]]	622.0294	-57.07	-5.69576	-0.00167	-5.0708
5	2VZ6	1	5	-7.40146	3.09678	2 3 17 7	[[28,36,43,66,3,8,19,50,51,52],[8,19,27,28,50,51,52,56,66,2],17,[34,84,26,29,75,76,91,54,57,67],[77,30,37,44,76,93,92,36,42,43],[68,23,39,62,11,15,21,31,32,63]]	151.9944	-96.6803	-5.52145	-26.0062	-7.40146
5	2VZ6	1	5	-7.31385	1.109239	2 3 4 5 7	[[53,88,89,10,17,27,34,63,4,7],[4,10,11,27,34,53,54,63,67,84],[4,9,13,20,21,22,26,29,30,34],[34,84,26,27,75,89,91,18,35,40],[51,3,50,52,55,58,66,1,2,5],[35,41,43,90,94,36,40,42,45,88],30,30,24,[39,85,32,38,86,23,87,64,83,6]]	159.7499	-104.649	-8.60217	-17.6054	-7.31385

5	2VZ6	1	5	-7.30258	2.855572	2 3 17 1[illegible]	[[28,36,43,66,8,19,50,51,52,56],[8,19,27,28,50,52,56,66,81,83],17,17,34,34,[34,26,29,75,76,84,93,37,54,57],[37,44,76,77,91,92,36,42,45,46],[68,21,60,62,86,11,12,15,23,31,68,21,60,62,86,11,12,15,23,31]]	140.7892	-72.3753	-5.33869	-31.2666	-7.30258
5	2VZ6	1	5	-7.04057	3.287058	2 12 13 1[illegible]	[[21,67,68,11,12,60,4,5,13,15,21,67,68,11,12,60,4,5,13,15],[51,58,66,77,3,9,19,20,30,8],[2,5,50,51,55,61,69,22,6,12],[81,33,82,83,24,65],[94,36,43,42,45,52,66,56,72,74]]	85.68433	-42.9952	-5.28463	-33.0935	-7.04057
5	2VZ6	1	5	-6.90586	3.789803	2 3 5 6 [illegible]	[[28,36,43,66,3,8,19,50,51,52],[8,19,27,28,50,52,56,66,2,3],[46,48,95,97,28,36,42,45,47,49],34,34,[34,26,29,75,76,84,88,93,54,57],[77,30,37,44,76,91,36,42,43,45],[68,15,63,87,11,21,23,31,62,78]]	148.9113	-66.4812	-6.29196	-24.8039	-6.90586

6	2VZ6	1	6	-8.42855	2.38433	2	3	45	4	[[35,76,33,34,74,75,30,31,32,73],[5,33,35,74,76,26,30,31,36,55],[30,32,34,35,33,31],[5,19,20,23,24,26,36,40,62,65],[1,37,38,90,91,30,83,2,29,45],[2,74,33,35,76,4,29,31,34,75]]	957.2028	-71.4028	-8.64397	-36.6363	-8.42855
6	2VZ6	1	6	-8.34246	6.298863	2	3	19	5	[[73,81,1,30,32,37,39,40,77,23],[1,5,39,62,68,81,6,19,20,22],[3,5,6,18,22,26,41,42,54,62],[5,33,35,74,76,30,31,34,75,26],[55,91,11,66,78,2,29,45,71,92],[2,30,31,32,33,34,35,73,74,75],[43,44,56,87,88,100,12,50,57,8]]	1070.721	-29.2511	-6.84503	-43.1326	-8.34246
6	2VZ6	1	6	-7.25351	3.814218	2	3	43	17	[[2,30,31,33,77,1,4,29,32,34],[1,2,29,31,37,77,4,20,30,36],43,38,[1,37,38,62,67,4,19,20,24,26],[5,26,71,4,27,45,90,92,70,80],[25,68,69,6,82,85,86,7,22,23]]	857.5286	-52.0341	-8.86745	-44.4517	-7.25351

6	2VZ6	1	6	-7.09415	5.898933	19 4 7 26	[[42,61,64,83,84,85,6,18,21,22],[3,41,42,61,82,83,84,85,86,6],[84,86,5,26,39,40,70,80,81,6],[74,33,35,76,2,4,26,27,28,29],[40,70,72,78,79,81,28,65,80,1],[40,58,65,79,81,14,23,57,7,9]]	960.2541	-26.5369	-5.12703	-38.5379	-7.09415
6	2VZ6	1	6	-6.91705	3.242656	2 3 17 18	[[86,82,83,41,42,20,38,54,62,72],[38,41,42,54,70,82,83,85,86,11],38,38,[4,5,26,30,31,32,33,34,35,37],[40,79,80,39,65,81,93,95,7,23],[2,29,45,90,92,4,28,72,91,30],[43,88,100,66,67,44,87,89,50,57,43,88,100,66,67,44,87,89,50,57]]	942.295	-25.415	-5.41367	-35.3665	-6.91705
7	2VZ6	1	7	-7.0245	1.76349	2 3 4 5 7	[[81,36,49,71,80,10,17,25,32,33],[4,10,11,26,32,36,49,50,58,65],[9,13,21,22,29,40,50,53,63,65],[32,79,83,81,26,35,36,73,80,82],[47,3,9,46,48,51,54,62,1,2],[28,37,48,87,62,27,33,34,38,42],24]	171.007	-69.9958	-6.74987	-15.2834	-7.0245

7	2VZ6	1	7	-6.8647	3.516333	2	3	17	18	[[28,34,39,62,3,8,19,46,47,48],[8,19,27,28,46,47,48,52,62,2],17,17,[7,17,25,27,33,37,46,48,52,60],[42,44,87,89,28,34,37,38,41,43],[32,79,26,29,73,74,81,82,85,50],[75,30,35,40,74,83,84,34,38,39],[63,23,57,69,11,14,21,58,12,15]]	189.6399	-95.0282	-8.21401	-23.7254	-6.8647
7	2VZ6	1	7	-6.71457	1.315774	2	3	33	34	[[62,3,12,19,46,47,48,51,55,1],[3,8,19,46,47,48,55,62,1,2],17,17,[2,7,10,17,18,25,27,33,36,37],[19,28,34,38,39,42,62,87,8,48],29,[63,79,4,21,32,49,50,53,74,10],[38,42,44,45,43,41],[38,42,44,45,43,41],[40,42,44,45,83,84,85,87,89,90],13,[31,76,78,23,57,58,69,77,13,14]]	199.3567	-122.943	-7.35301	-13.9509	-6.71457

7	2VZ6	1	7	-6.64528	2.77781	2	3	19	33	[[62,3,19,47,48,51,1,5,8,9],[3,8,19,46,47,48,51,62,1,2],[1,2,3,6,7,8,16,17,18,24],17,17,[2,7,10,17,18,25,27,33,36,37],[19,28,34,38,39,42,62,87,8,48],29,[65,4,21,32,49,50,53,74,79,10],[38,42,44,45,43,41],[38,42,44,45,43,41],[40,42,44,45,83,84,85,87,89,90],[69,23,57,56,12,14,15,21,55,63]]	185.6661	-103.823	-7.87511	-9.0705	-6.64528
7	2VZ6	1	7	-6.59226	1.404911	2	3	17	18	[[28,34,38,42,87,39,41,43,44,62,28,34,38,42,87,39,41,43,44,62],[8,27,37,48,52,70,2,7,17,18],17,17,[80,81,36,63,72,79,82,65,25,49],32,[64,69,13,22,23,67,68,4,5,11]]	144.8563	-52.6394	-7.24365	-31.6118	-6.59226
8	2VZ6	1	8	-7.31206	2.254784	3	19	4	37	[[79,11,49,70,77,85,86,9,15,19],[9,11,15,19,23,49,77,79,85,86],[8,9,10,11,12,15,19,25,30,33],[57,58,66,4,62,1,56,80,7,74],[38,30,92,12,19,44,55,80,10,11],[4,48,67,81,82,84,13,22,65,14],[48,83,84,14,46,20,32,36,68,5]]	460.2786	-36.0695	-5.82915	-36.0986	-7.31206

8	2VZ6	1	8	-6.88562	3.60046	3	19	4	37	[[79,9,11,49,70,77,85,86,19,5],[9,11,15,19,23,49,77,79,85,86],[8,9,10,11,12,15,19,25,30,33],[57,58,62,66,1,56,80,7,74,75],[38,12,19,30,44,80,8,10,11,78],[48,83,84,14,46,20,32,39,67,68]]	460.377	-24.1773	-4.18686	-32.0273	-6.88562
8	2VZ6	1	8	-6.7933	2.171338	2	3	19	7	[[15,51,75,80,85,86,12,23,25,33],[7,12,15,44,51,54,75,80,85,86],[7,19,28,29,30,35,38,44,54,55],[92,39,14,23,32,42,49,83,84,8],[81,13,37,48,53,73,20,21,22,27]]	498.1541	-47.1393	-4.50769	-25.4151	-6.7933
8	2VZ6	1	8	-6.33891	3.259934	3	19	4	38	[[8,9,10,11,76,77,78],[10,12,78,80,86,8,9,11,15,19],[10,12,15,51,74,78,80,85,86,8],[7,25,51,74,75,45,85,86,52,6],[9,77,8,11,79,76,10,12,19,78],[8,10,12,19,11,9],[4,41,48,65,66,22,82,55,84,92],[65,84,92,14,39,83,29,32,38,48]]	491.247	-17.814	-6.83294	-32.2885	-6.33891

8	2VZ6	1	8	-6.31932	2.600005	3 19 4 3[illegible]	[[78,10,12,15,70,80,85,86,8,49],[10,12,15,19,44,78,80,85,86,23],[8,9,10,11,12,19,30,38,44,54],[7,54,58,63,74,75,1,56,57,3],[9,77,8,76,11,19,39,79,92,10],[14,46,48,83,84,20,22,24,32,42]]	483.3174	-12.526	-6.41286	-29.5139	-6.31932
9	2VZ6	1	9	-5.45993	2.319699	2 13 14	[[10,44,37,47,73,1,3,4,8,21,10,44,37,47,73,1,3,4,8,21],[16,27,45,63,64,74,31,6,11,17],[28,66,67,18,51,65,48,49,17,53]]	83.20825	-48.531	-5.49516	-25.6256	-5.45993
9	2VZ6	1	9	-5.37063	3.345113	2 3 13 1[illegible]	[[43,50,68,9,14,30,40,42,75,1,43,50,68,9,14,30,40,42,75,1],[14,43,46,50,13,47,51,5,24,58],[8,10,19,29,38,44,52,69,73,74],[72,74,31,34,35,56,21,73,23,45]]	88.06138	-37.1881	-5.26349	-10.9562	-5.37063
9	2VZ6	1	9	-5.28771	0.912127	3 17 18 [illegible]	[[15,21,26,27,29,31,35,38,63,64],26,26,[2,6,7,11,15,16,17,18,21,26],[68,75,25,30,33,36,61,62,77,20],[41,42,58,10,47,3,5,9,13,44]]	107.7354	-46.7141	-8.33569	-9.35502	-5.28771
9	2VZ6	1	9	-5.09776	3.680158	2 13 14 [illegible]	[[10,44,38,52,71,3,8,9,19,42],[26,29,32,36,62,70,75,69,15,25],[16,27,63,64,65,6,26,45,48,55],35]	76.09432	-31.1186	-5.20824	-24.3054	-5.09776

9	2VZ6	1	9	-4.91579	2.293516	2 3 17 1[illegible]	[[61,68,75,77,30,36,43,2,14,25],[2,7,16,18,25,40,43,50,61,62],77,[53,54,13,22,46,47,51,60,23,49],[31,34,72,73,21,35,37,55,56,74]]	94.3508	-54.7514	-7.2184	-20.233	-4.91579
10	2VZ6	1	10	-6.61412	6.321403	2 3 13 6	[[7,51,76,77,23,34,38,46,1,24,7,51,76,77,23,34,38,46,1,24],[11,18,38,76,92,8,9,10,12,78],[5,17,36,43,47,53,71,72,61,63],40,[69,85,86,4,49,67,14,19,32,40]]	429.9716	-57.8	-4.7869	-30.3904	-6.61412
10	2VZ6	1	10	-6.49719	5.123814	2 3 43 4[illegible]	[[9,11,38,79,81,92,8,10,12,18],[9,11,38,76,79,81,92,7,23,27],36,36,[1,7,15,23,24,26,35,51,52,76],[58,59,60,1,64,3,4,16,67,68],[42,49,84,13,14,21,56,83,85,86]]	428.7578	-66.4168	-7.97151	-30.3161	-6.49719
10	2VZ6	1	10	-6.28821	4.451087	28 2 3 1[illegible]	[[60,1,51,52,58,59,23,24,34,35],[1,51,59,60,23,27,34,38,55,77],[1,7,51,59,60,77,8,9,10,11],[6,29,44,57,73,74,75,32,40,93],[93,40,39,69,4,42,67,68],[3,64,65,66,69,81,39,41,89,9]]	412.545	-63.0413	-5.83993	-32.247	-6.28821

10	2VZ6	1	10	-6.1882	2.260586	2	32	13	1	[[1, 7, 58, 76, 77, 12, 18, 38, 51, 54, 1, 7, 58, 76, 77, 12, 18, 38, 51, 54], [8, 10, 12, 18, 11, 9], [6, 40, 57, 73, 74, 75, 82, 12, 29, 30], [86, 40, 93, 67, 68, 4, 14, 42, 49, 21], [84, 13, 48, 90, 42, 64, 3, 4, 16, 41]]	417.8348	-20.0503	-6.28022	-30.3411	-6.1882
10	2VZ6	1	10	-6.15136	4.636907	2	3	19	7	[[8, 10, 12, 38, 80, 82, 92, 9, 11, 18], [10, 12, 38, 80, 82, 92, 7, 51, 76, 77], [1, 7, 23, 35, 51, 52, 59, 76, 77, 80], [58, 66, 1, 59, 60, 3, 41, 65, 67, 68], [21, 42, 49, 67, 84, 4, 13, 56, 69, 83]]	449.2291	-60.6026	-7.83661	-22.8998	-6.15136
11	2VZ6	1	11	-8.74178	2.067727	4	7	26	27	[[9, 17, 30, 32, 37, 40, 58, 59, 84, 85], [61, 62, 1, 82, 40, 55, 85, 9, 27, 56], [72, 5, 73, 80, 12, 53, 74, 88, 93, 94], [65, 83, 2, 8, 18, 38, 47, 63, 64, 57]]	514.2079	4.347256	-4.47316	-42.3152	-8.74178
11	2VZ6	1	11	-8.02371	3.692272	2	3	19	5	[[72, 5, 12, 15, 44, 65, 88, 93, 94, 22], [12, 48, 72, 85, 88, 94, 15, 19, 22, 30], [9, 10, 19, 30, 31, 32, 37, 40, 48, 58], [11, 21, 43, 71, 10, 12, 19, 22, 44, 4], 41, 41, [100, 41, 34, 91, 92, 14, 32, 40, 59, 77], [44, 73, 74, 4, 5, 21, 22, 43, 70, 72], 46, [50, 8, 47, 57, 82, 83, 89, 99, 13, 20]]	487.392	-69.2316	-4.10342	-43.3362	-8.02371

11	2VZ6	1	11	-7.95438	2.408828	3	19	4	5	[[44,72,73,5,22,53,80,88,93,94],[12,15,25,53,88,93,94,19,22,32],[10,12,15,19,27,32,35,40,48,55],[43,70,4,71,11,21,69,87],[74,88,100,10,11,12,19,21,22,69],[77,91,92,14,20,34,50,52,78,7]]	501.9652	-71.262	-8.80191	-41.5136	-7.95438
11	2VZ6	1	11	-7.40843	2.595442	2	13	14	8	[[12,19,61,85,94,1,15,27,32,35,12,19,61,85,94,1,15,27,32,35],[14,31,34,41,59,75,76,92,100,86],[75,77,6,45,66,76,90,98,13,24],[69,73,4,5,11,12,21,22,43,44],[68,97,3,17,84,96,66,42,67,98]]	489.0629	-52.7374	-4.22717	-42.913	-7.40843
11	2VZ6	1	11	-7.1626	4.055127	28	2	3	7	[[66,3,67,9,61,84,85,1,42,60],[9,61,66,84,85,1,58,62,72,5,9,61,66,84,85,1,58,62,72,5],[10,19,31,32,48,58,59,85,86,34],[53,79,93,94,40,7,25,46,78,80],[4,11,21,22,43,69,70,87,44,71],[7,50,78,80,92,57,82,8,47,14]]	492.2948	-38.1091	-4.78017	-33.5777	-7.1626

12	2VZ6	1	12	-7.38521	2.179604	2	32	3	17	[[92,13,21,32,86,30,40,47,53,57,92,13,21,32,86,30,40,47,53,57],[9,11,13,21,12,10],[8,30,37,53,57,80,81,96,18,42],42,42,42,42,[9,11,13,21,12,10],[41,74,97,6,76,89,90,15,34,44],[64,2,7,19,46,56,63,78,79,87]]	332.6437	-37.3904	-3.39773	-43.9528	-7.38521
12	2VZ6	1	12	-6.88762	3.352767	2	3	19	5	[[11,13,16,84,86,91,92,25,35,47],[13,16,47,86,91,92,21,26,31,32],[21,26,30,31,32,40,47,53,57,58],[9,82,10,11,13,21,84,83,12,85],41,41,[41,97,21,32,40,58,85,15,34,90],[9,11,82,84,10,13,83,86],44,[7,51,56,77,78,87,14,15,22,24,7,51,56,77,78,87,14,15,22,24]]	334.2698	-72.8813	-6.98737	-38.0733	-6.88762
12	2VZ6	1	12	-6.7606	3.317441	4	37	7	11	[[11,12,13,16,21,27,32,35,40,47],[59,60,71,72,1,12,40,61,85,8],[97,9,10,12,13,21,32,40,47,58],[51,89,90,15,49,22,34,39,74,6]]	457.0801	-26.6229	-3.57295	-31.7107	-6.7606

12	2VZ6	1	12	-6.72075	3.003134	2 3 43 4[illegible]	[[1,61,80,8,81,13,54,65,84,86],[1,8,13,54,57,61,80,81,86,11],42,42,40,[12,21,31,32,40,47,57,58,85,13],40,[52,76,10,83,9,11,12,82,85,21],[75,49,89,6,15,22,44,51,53,74]]	461.8106	-49.1602	-3.71885	-32.2796	-6.72075
12	2VZ6	1	12	-6.43642	1.83513	2 3 4 5 [illegible]	[[19,38,46,49,56,87,7,39,45,65],[14,22,23,46,49,56,87,88,15,24],[5,15,22,24,26,31,32,34,36,40],[14,71,87,88,23,46,50,63,70,95],[54,80,8,27,28,30,37,55,57,81],[62,64,77,78,93,94,2,19,38,42],[6,75,76,25,44,52,74,91,3,16]]	517.497	-65.8664	-5.08276	-1.25636	-6.43642
13	2VZ6	1	13	-6.724	2.31456	2 3 17 1[illegible]	[[29,37,76,27,43,46,47,53,20,21],[19,20,21,27,29,37,40,43,46,47],40,40,[5,14,19,24,32,35,40,41,44,45],[53,83,11,27,29,31,38,43,72,77],36,[48,56,1,22,23,33,49,52,84,88],[36,88,52,71,7,8,9,10,11,18]]	395.9431	-48.4179	-6.84102	-10.1311	-6.724

13	2VZ6	1	13	-6.4095	3.016065	2	3	45	17	[[7,8,9,10,11,18,73,74,76],[9,11,18,36,54,75,77,88,7,8],[7,9,11,18,10,8],36,36,[26,28,36,52,54,77,88,11,33,42],36,36,[22,26,33,35,36,48,51,52,59,60],[9,75,46,51,64,11,47,60,77,4],[4,40,65,66,83,14,38,82,19,27]]	314.2041	-55.1717	-6.40944	-28.7091	-6.4095
13	2VZ6	1	13	-6.40064	2.243903	2	3	13	54	[[78,79,2,12,34,46,58,59,13,15,78,79,2,12,34,46,58,59,13,15],[37,8,10,18,74,76,7,9,11,29],[48,49,60,84,63,2,17,22,35,1],35,35,[55,56,1,17,35,52,44,57,26,36],[61,5,67,68,69,86,3,49,63,1],[71,6,54,70,42,72,28,31,38,89]]	312.032	-14.126	-5.74363	-31.7212	-6.40064
13	2VZ6	1	13	-6.21035	5.500666	3	19	4	7	[[75,77,88,9,11,26,36,52,54,72],[9,11,18,26,29,36,43,75,77,88],[2,8,9,10,11,18,29,37,43,51],[89,8,74,7,73,10,18,38,76,9],[14,45,82,83,19,47,21,31,40,50]]	317.1322	-62.4051	-6.93878	-28.943	-6.21035

13	2VZ6	1	13	-6.20651	1.902437	2	3	17	3[illegible]	[[37,53,27,29,46,51,83,59,79,10],[14,27,37,38,46,53,83,12,19,20],40,40,[1,4,5,14,19,20,24,32,40,41],[83,89,38,14,31,53,71,72,77,27],36,36,[48,22,23,33,35,49,52,60,84,88],[36,88,7,8,9,10,11,18,29,43],[54,70,71,6,28,42,52,56,57,72]]	420.5271	-62.7977	-5.44437	-8.15482	-6.20651
14	2VZ6	1	14	-7.1235	6.621377	19	4	38	2[illegible]	[[14,44,8,84,3,39,72,85,83,25],[3,64,84,8,14,27,56,63,4,11],[3,27,39,57,64,65,83,84,85,4],[98,100,49,78,99,6,42,60,2,23],[61,73,78,9,34,46,67,74,92,2]]	136.7448	-52.767	-5.94754	-38.6903	-7.1235
14	2VZ6	1	14	-6.50751	8.851389	19	33	34	[illegible]	[[9,23,34,37,46,56,61,74,79,92],9,9,[64,71,72,3,4,27,33,57,63,8]]	267.8622	-41.5717	-3.44257	-29.4144	-6.50751
14	2VZ6	1	14	-6.36838	5.041188	28	2	13	1[illegible]	[[92,46,93,73,78,2,9,37,61,79],[73,92,78,79,106,2,34,37,53,55],[12,41,48,51,52,54,105,107,55,5],[47,95,96,97,4,40,63,83,85,26],[11,12],12,[38,80,81,82,71,62,85,91,25,33]]	318.2392	-46.7473	-3.70134	-34.6358	-6.36838

14	2VZ6	1	14	-6.24584	4.857532	2 13 54 5	[[37,77,79,100,2,49,65,98,6,42,37,77,79,100,2,49,65,98,6,42],[13,7,32,42,43,66,70,86,88,29],7,7,[86,7,43,87,88,64,4,26,27,32],[101,102,50,103,39,84,85,3,8,14,101,102,50,103,39,84,85,3,8,14]]	313.2203	-34.9966	-4.42194	-33.1139	-6.24584
14	2VZ6	1	14	-6.15673	8.726407	28 2 13 1	[[99,49,98,100,37,77,78,79,2,61],[77,78,99,37,60,61,74,90,92,2],[69,73,102,103,8,9,24,31,34,44],[101,33,50,71,72,84,102,103,39,83],14,14,[51,104,12,41,48,53,55,106,65,5,51,104,12,41,48,53,55,106,65,5]]	172.0919	-23.0366	-4.7661	-34.4569	-6.15673
15	2VZ6	1	15	-7.71648	1.729031	2 3 5 7 8	[[49,58,65,67,7,19,25,26,28,43],[7,26,43,49,60,61,65,1,8,10],[3,6,29,43,65,13,20,49,50,72],[45,12,15,39,42,44,51,53,68,11],[2,18,47,48,66,4,38,28,33,35],[31,33,35,36,34,32],[4,6,32,38,71,29,31,34,73,69],[27,55,62,63,64,44,46,7,12,15]]	296.8552	-71.558	-8.06922	-23.562	-7.71648

15	2VZ6	1	15	-7.68466	1.385858	2 3 5 7 8	[[49,58,63,65,67,7,19,25,26,27],[7,26,27,43,49,60,63,65,1,8],[3,6,29,43,65,13,20,49,50,72],[45,12,15,39,42,44,51,53,68,11],[2,18,47,48,66,4,28,33,35,65],[31,33,35,36,34,32]]	285.8168	-54.2641	-7.84644	-24.5309	-7.68466
15	2VZ6	1	15	-7.43884	1.842902	2 3 7 8 3	[[49,58,65,67,7,19,25,26,28,43],[7,26,43,49,61,62,65,1,8,10],[45,12,15,39,42,44,51,53,68,11],[66,2,18,38,47,48,4,6,28,29],[31,33,35,36,34,32]]	303.8353	-94.0647	-8.3974	-21.4634	-7.43884
15	2VZ6	1	15	-7.11505	2.896673	1 2 3 4 7	[20,[1,5,6,20,37,50,52,3,13,14],[1,3,6,13,20,37,43,50,14,29],[2,7,8,10,19,23,25,26,28,43],[47,9,12,18,39,42,48,56,59,4],[73,32,34,36,75,71,31,6,4,3]]	277.3485	-64.8237	-8.55059	-23.8996	-7.11505
15	2VZ6	1	15	-7.05322	1.119027	1 41 2 13	[1,1,[1,3,6,13,20,29,37,43,50,60,1,3,6,13,20,29,37,43,50,60],[4,9,12,17,18,39,42,45,47,48],[70,4,38,47,68,29,31,32,33,34]]	286.5275	-86.8352	-8.14407	-29.2781	-7.05322

16	2VZ6	1	16	-7.54022	3.863869	2	3	19	4	[[64,92,1,14,17,57,87,90,97,98],[1,9,14,49,57,60,63,64,87,92],[6,9,23,32,33,34,39,43,49,60],[3,6,21,34,41,43,49,61,69,77],[10,12,14,23,13,11],[7,36,46,50,68,82,16,27,55,80],[82,81,10,12,88,90,11,89,7,80],[93,15,48,54,59,25,26,31,52,85,93,15,48,54,59,25,26,31,52,85]]	346.5084	-48.1639	-2.06745	-28.3616	-7.54022
16	2VZ6	1	16	-7.05873	2.553166	2	32	13	1	[[55,97,98,12,14,17,23,27,49,90,55,97,98,12,14,17,23,27,49,90],[10,12,14,23,13,11],[43,75,79,101,13,91,5,19,23,34],[75,5,66,67,76,100,74,2,18,19],45,45,[79,77,74,6,44,45,78,94,15,26]]	311.148	-13.9305	-3.90597	-43.7127	-7.05873
16	2VZ6	1	16	-6.22707	3.891316	28	2	13	1	[[4,71,72,22,42,52,84,8,47,51],[52,84,24,54,59,93,95,8,26,31],[20,25,40,44,53,76,94,5,15,19],[5,74,75,76,77,44,6,78,79,19],43,43,[41,13,91,11,14,23,34,89,43,21]]	308.8383	-27.684	-1.47734	-34.982	-6.22707

16	2VZ6	1	16	-6.09373	2.369942	2 3 33 3[illegible]	[[63,89,91,86,1,13,62,9,11,23],[1,9,11,13,14,23,32,34,39,49],42,42,[4,8,18,22,29,30,37,39,42,47],[9,86,87,63,1,5,39,44,64,75],[52,95,16,24,47,54,56,59,96,28],[80,82,7,81,27,46,50,55,69,17]]	395.6304	-13.2026	-1.68	3.157182	-6.09373
16	2VZ6	1	16	-6.01965	4.914754	2 3 17 1[illegible]	[[52,71,95,56,4,16,24,47,51,72],[24,47,52,54,59,93,95,8,15,26],48,48,[2,20,25,40,48,53,65,66,67,93],[85,87,8,9,58,66,83,86,84,2],[1,62,64,5,44,75,76,9,19,39,1,62,64,5,44,75,76,9,19,39],[10,12,14,23,13,11]]	333.587	-32.6855	-2.66752	-22.5117	-6.01965
17	2VZ6	1	17	-6.98415	1.936173	2 3 4 5 7	[[51,70,8,9,17,29,30,57,68,73],[3,8,9,30,47,51,57,60,68,70],[7,13,16,22,26,27,32,46,47,49],[30,73,23,45,68,77,14,24,51,37],[42,2,4,43,44,48,50,55,1,5],27,27,28,[61,76,19,20,28,33,53,62,63,75]]	207.4409	-71.0803	-7.50531	-23.4347	-6.98415

17	2VZ6	1	17	-6.65188	1.443182	2 3 4 5 6	[[51,68,72,8,9,21,24,29,30,57],[3,8,9,23,30,47,51,57,60,68],[7,13,18,22,26,36,46,47,49,58],[30,73,79,23,32,45,68,14,24,26],[34,38,40,41,39,37],[42,1,2,5,43,44,48,50,4,6],[44,70,81,25,55,24,31,34,38,40]]	225.605	-110.844	-8.69082	-12.6513	-6.65188
17	2VZ6	1	17	-6.41208	2.284023	2 3 17 4	[[79,46,66,7,16,26,27,32,36,47],[13,18,26,30,32,36,46,47,49,58],30,[3,8,9,11,17,18,21,29,51,56],28,28,[43,2,4,6,11,42,44,52,63,1],[48,54,65,67,12,53,5,22,66,13],[38,40,81,83,34,37,25,31,45,24],[34,38,40,41,39,37]]	213.6538	-84.6822	-8.05098	-25.5039	-6.41208
17	2VZ6	1	17	-6.3428	1.812543	2 3 4 48	[[38,40,81,83,34,41,79,84],[38,81,15,25,31,34,35,40,55,79],[2,6,15,24,25,31,35,42,43,44],30,30,[68,45,73,37,39,41,80,82,24,51],[58,46,67,13,26,30,32,47,49,36]]	200.2595	-47.7234	-7.69956	-25.5537	-6.3428

17	2VZ6	1	17	-6.17898	1.631527	2 3 45 12	[[73,14,23,24,30,45,51,68,8,78,73,14,23,24,30,45,51,68,8,78],[39,40,41,83,84,34,37,38,82],[34,38,40,41,39,37],[70,72,29,43,64,71,75,4,11,20],[2,6,35,42,43,44,55,52,15,25],[54,53,5,12,42,43,48,13,19,62]]	188.9334	-109.779	-7.96557	-29.2453	-6.17898
18	2VZ6	1	18	-6.30995	3.155664	2 3 5 6 2	[[31,49,2,34,38,45,7,10,32,52],[2,10,14,31,32,34,44,45,49,52],[44,45,6,14,15,16,42,47,49,2],24,24,[56,57,3,8,23,33,36,55,61,63],[18,39,48,50,51,60,25,6,16,22]]	756.9987	-68.8773	-7.51125	-15.6186	-6.30995
18	2VZ6	1	18	-6.26746	3.563006	3 33 34 4	[[3,8,23,25,33,36,50,57,59,9],13,13,[4,5,11,13,20,21,27,30,35,36],[4,10,19,31,32,34,38,52,2,7],[46,15,42,44,47,2,6,14,16,22]]	421.1323	-56.3414	-7.10535	-24.4154	-6.26746
18	2VZ6	1	18	-6.19427	1.519622	2 3 4 5 6	[[31,49,2,34,38,45,7,10,32,39],[2,10,14,31,32,34,44,45,49,52],[4,5,11,13,21,32,37,40,41,53],[45,44,6,14,15,16,31,42,47,49],24,24,[56,57,3,8,23,33,36,61,63,17],[18,33,39,48,50,51,60,25,6,16]]	757.2541	-69.7856	-7.82523	-14.5658	-6.19427

18	2VZ6	1	18	-5.36194	1.194003	2 3 6 20	[[14,31,44,45,49,2,6,16,32,34],[1,2,4,5,14,31,32,34,37,43],24,24,[33,56,57,3,8,23,36,59,7,17],[22,18,39,46,50,51,61,6,15,16],[68,30,29,62,67,69,24,26,58,19]]	789.3881	-78.5848	-7.69112	-10.6414	-5.36194
18	2VZ6	1	18	-5.28275	1.739983	2 3 7	[[50,18,51,61,3,9,23,25,36,57],[3,9,18,23,33,36,50,51,56,57],[32,66,1,2,4,28,31,34,37,38]]	744.3671	-69.9888	-8.28027	-4.75918	-5.28275
19	2VZ6	1	19	-6.30484	3.584318	2 3 17 7	[[28,33,36,59,3,8,19,43,44,45],[8,19,28,43,44,45,49,59,71,2],17,[32,77,26,29,70,72,80,47,50,60],[73,30,34,37,72,78,33,35,36,38],[61,23,54,66,11,14,21,55,53,12]]	212.4279	-105.192	-7.15301	-19.2075	-6.30484
19	2VZ6	1	19	-6.22322	1.998948	2 3 4 5 7	[[46,69,71,10,11,17,25,27,32,55],[4,10,11,26,32,46,47,55,62,69],[9,13,22,29,37,47,50,60,64,65],[32,77,80,26,34,70,18,27,29,46],[44,3,43,45,48,51,59,1,2,5],[45,67,82,28,59,27,33,35,39,69],[66,23,75,74,15,16,24,31,56,58]]	193.2816	-94.647	-7.10536	-9.67208	-6.22322

19	2VZ6	1	19	-6.10184	2.57528	2	3	33	38	[[39,41,42,82,84,33,35,38,40,81],[39,41,82,84,27,28,35,70,80,33],32,[21,25,32,46,60,62,67,68,69,10],[37,78,79,34,73,29,72,80,77,20],[48,51,9,44,52,59,1,3,5,8]]	187.6161	-57.7349	-7.57912	-28.8531	-6.10184
19	2VZ6	1	19	-6.01076	0.989188	2	12	13	1	[[43,45,49,69,2,7,8,44,58,76],[41,84,27,39,42,71,82,32,70,78],[27,32,46,62,67,71,77,25,68,7],[61,21,54,60,62,14,66,11,23,55],32,32,[78,84,26,32,34,37,70,77,79,29]]	172.1992	-62.3697	-8.01634	-34.8303	-6.01076
19	2VZ6	1	19	-6.00393	2.772542	2	3	5	6	7	[[23,53,66,5,12,13,47,50,64,1],[4,11,13,21,23,47,50,61,66,72],[21,61,11,14,23,54,62,66,76,15],19,[45,49,3,7,8,25,27,43,44,59],[52,12,16,24,43,56,58,57,6,15],[36,81,33,35,37,38,79,80,19,20]]	221.0561	-128.058	-8.45247	-9.56061	-6.00393

20	2VZ6	1	20	-7.24016	3.908734	1 2 3 33	[12,[41,55,12,20,21,27,40,10,11,13],[12,19,21,27,40,41,55,10,11,13],30,[6,8,17,18,19,30,35,37,38,39],48,48,[58,15,18,31,48,82,88,89,16,32],[70,46,87,42,43,44,45,84,85,40],[42,47,46,45,44,43],[73,74,28,29,33,67,22,23,24,61]]	172.915	-76.2276	-7.01065	-28.4006	-7.24016
20	2VZ6	1	20	-7.10836	1.625477	2 3 4 5 7	[[89,26,37,38,75,82,9,18,30,35],[9,15,22,24,26,37,38,48,61,63],[14,16,28,29,32,33,56,59,61,66],[48,71,89,15,31,38,58,7,16,37],[21,10,17,19,53,55,57,4,5,6],[81,19,27,83,88,55,18,37,39,40],34]	177.9036	-75.4021	-7.37989	-16.2239	-7.10836
20	2VZ6	1	20	-7.06188	1.159786	2 12 13 1	[[19,11,54,77,81,7,8,10,12,17,19,11,54,77,81,7,8,10,12,17],[87,38,46,83,37,39,45,47,58,82],[26,37,38,48,74,76,82,89,39,9],[73,33,62,72,74,23,24,51,63,2],[52,51,2,64,80,36,1,3,25,34]]	144.472	-60.3085	-7.70365	-38.8056	-7.06188

20	2VZ6	1	20	-7.00619	1.853223	2 12 40 1	[[19,11,21,54,55,75,81,7,8,10,19,11,21,54,55,75,81,7,8,10],[87,46,39,45,47,58,71,82,83,86],38,[26,37,38,48,74,89,82,7,9,15],[73,33,62,72,74,24,63,23,51,2],[52,51,2,25,34,64,80,36,1,3]]	148.9106	-75.7355	-8.55902	-35.5595	-7.00619
20	2VZ6	1	20	-6.8885	1.706525	2 13 14 1	[[19,11,17,21,54,55,77,7,8,10,19,11,17,21,54,55,77,7,8,10],[26,37,39,73,81,82,83,38,9,30],[72,33,62,73,74,63,23,3,24,51],[51,2,49,52,64,80,1,50,25,34]]	153.1002	-42.0407	-7.1302	-37.1581	-6.8885
21	2VZ6	1	21	-8.79148	2.175096	2 3 4 5 7	[[94,95,98,45,47,43,96,49,99,97],[35,45,79,94,95,16,26,28,31,62],[6,8,9,15,16,20,28,31,34,35],[35,42,45,46,94,99,49,98,97,37],[69,48,67,73,37,40,41,44,91,92],[91,96,97,39,42,45,46,47,49,98],[11,13,23,51,58,74,5,55,57,1]]	146.6967	-71.0575	-8.42777	-41.5502	-8.79148

21	2VZ6	1	21	-7.92871	2.671835	2 3 17 12[illegible]	[[6,8,15,18,25,56,61,77,79,81,6,8,15,18,25,56,61,77,79,81],[56,61,79,81,34,48,31,37,38,40],48,[87,36,53,88,89,30,33,76,57,71],[17,53,54,55,57,64,65,72,73,1],[72,74,12,23,59,66,73,13,60,5],[41,44,92,93,38,90,40,91,37,46],[11,22,53,57,58,71,73,30,33,87]]	144.1324	-67.7891	-6.01392	-48.922	-7.92871
21	2VZ6	1	21	-7.64622	2.772712	3 17 18 [illegible]	[[46,97,99,35,37,39,42,49,34,40],35,35,35,35,[20,28,31,35,43,47,48,62,68,69],[94,95,43,48,45,47,81,39,42,96],[32,70,84,10,21,29,51,52,4,22],[83,27,50,82,90,92,73,38,40,41]]	151.8187	-44.2455	-5.95785	-43.8523	-7.64622
21	2VZ6	1	21	-7.5398	1.671246	2 3 17 18[illegible]	[[60,3,12,50,67,69,73,82,1,4,60,3,12,50,67,69,73,82,1,4],[40,46,69,34,37,39,42,44,91,93],46,46,[77,25,61,6,8,14,15,18,27,52],[6,8,15,56,61,63,79,83,18,27],[48,43,47,95,39,40,91,94,45,42],[83,90,92,38,41,27,81,82,44,93],[75,61,15,24,76,79,53,6,14,25]]	156.5533	-52.8484	-5.97291	-45.8336	-7.5398

21	2VZ6	1	21	-7.21365	4.172278	2 13 14 8	[[35,42,46,94,31,56,63,68,34,37,35,42,46,94,31,56,63,68,34,37],[7,26,48,55,62,66,74,78,79,80],[59,72,23,73,12,13,60,67,74,5],[48,43,96,40,91,97,37,39,42,45],[53,54,55,57,65,74,78,30,89,17]]	139.7352	-70.2042	-7.42188	-39.4332	-7.21365
22	2VZ6	1	22	-6.39868	3.805538	2 3 8	[[58,6,14,38,46,48,5,7,12,20],[12,14,20,23,38,43,56,58,59,63],[66,2,26,34,36,45,35,3,29,37]]	306.7271	-72.6069	-7.39098	-13.1868	-6.39868
22	2VZ6	1	22	-6.01522	2.357899	1 2 5 8	[26,[25,66,17,21,26,53,65,19,61,7],[51,25,9,16,17,43,52,60,61,65],[46,48,32,54,20,23,24,58,63,64]]	317.5465	-81.3089	-7.1292	-6.22448	-6.01522
22	2VZ6	1	22	-6.0035	3.761355	2 3 17 29	[[5,43,1,20,24,31,46,59,64,16],[20,43,46,58,59,64,4,6,12,14],30,[25,26],[25,26],[65,17,25,50,51,57,61,66,9,16],[53,10,21,26,65,66,44,47,54,5],[3,37,38,48,6,7,45,47,29,39]]	294.8612	-103.755	-7.85035	-23.0626	-6.0035

22	2VZ6	1	22	-5.88798	2.974981	2 3 5 8 3	[[58,6,14,39,46,48,7,23,44,56],[12,14,23,43,44,46,49,56,58,63],[41,49,8,11,12,30,34,39,50,55],[66,26,51,57,61,2,3,13,28,29],[2,13,28,34,35,36,52,49,11]]	345.0652	-88.8802	-7.31491	-12.4776	-5.88798
22	2VZ6	1	22	-5.63585	2.783762	2 3 4 29	[[48,54,43,5,7,10,44,63,19,20],[6,7,43,46,48,54,56,63,8,12],[3,6,8,11,12,14,29,37,39,45],25,[25,65,17,26,61,16,19,51,60,38],[34,2,27,35,36,40,62,67,22,31]]	370.5851	-89.1245	-9.14106	-14.0741	-5.63585
23	2VZ6	1	23	-5.55705	1.075281	2 3 17 18	[[52,59,60,62,21,25,27,33,44,53],[21,25,33,35,44,52,59,60,1,2],13,13,22,[31,38,8,11,22,29,54,4,6,14],[24,54,58,26,27,53,61,62,17,21],[49,20,42,43,47,48,50,51,10,15]]	61.50467	-82.1759	-8.42815	-16.6031	-5.55705
23	2VZ6	1	23	-5.51244	2.473422	2 3 17 18	[[27,52,59,60,62,21,23,25,33,53],[21,23,25,33,35,52,55,56,59,60],13,13,[31,38,8,11,22,29,54,4,6,14],[24,54,58,26,27,61,62,53,17,25],[49,15,20,42,43,50,7,16,37,44]]	61.46875	-70.1625	-8.80299	-14.5608	-5.51244

23	2VZ6	1	23	-5.42877	1.176842	3 4 6 20	[[25,33,35,52,59,2,6,9,14,16],[2,6,7,11,15,16,23,30,33,37],24,24,[31,3,4,8,28,29,38,46,48,51]]	84.09892	-50.2339	-8.53404	-11.3859	-5.42877
23	2VZ6	1	23	-5.38596	1.506028	2 3 17 18	[[52,59,62,21,25,33,45,53,2,6],[21,25,33,35,45,52,59,62,1,2],13,13,[31,38,8,11,22,29,54,4,6,14],[24,54,58,26,27,61,53,17,25,60],[49,15,20,42,43,47,50,51,10,37]]	57.90883	-63.8026	-8.57619	-14.2621	-5.38596
23	2VZ6	1	23	-5.21894	1.2928	2 3 17 33	[[62,21,25,26,33,44,52,53,59,14],[21,25,33,35,44,52,55,59,62,2],13,13,13,[46,8,17,24,28,31,58,3,18,32],[27,58,60,61,62,26,32,24,17,18],[20,50,51,37,49,4,10,11,29,38]]	58.21771	-66.9162	-8.17636	-18.2512	-5.21894
24	2VZ6	1	24	-6.61282	1.114819	2 3 5 7 8	[[33,46,56,60,69,80,3,8,9,14],[3,8,9,19,26,33,46,48,56,60],[33,80,26,35,70,81,27,30,46,39],[45,4,44,47,49,52,62,1,2,5],[67,37,47,50,71,84,25,27,34,39],[64,12,15,23,53,54,57,66,72,73]]	195.6278	-100.635	-6.7369	-14.9008	-6.61282

24	2VZ6	1	24	-6.29694	3.170795	2 3 4 7 2	[[40,42,85,87,36,43,88],[85,40,70,82,26,35,38,42,81,87],[8,18,19,26,27,33,46,60,61,70],[38,75,83,31,35,76,81,82,30,51],[49,52,64,22,53,54,63,5,11,45],[45,49,52,62,4,10,11,20,37,47]]	155.0572	-40.7031	-8.79208	-27.36	-6.29694
24	2VZ6	1	24	-6.27156	3.78036	3 19 4 7	[[36,39,40,41,42,43,85,86,87,88],[27,39,70,71,84,18,26,33,34,36],[18,26,27,33,35,39,41,70,80,84],[36,40,42,43,85,87,88,41,86,34],[45,62,4,10,11,20,44,47,49,52]]	145.5313	-73.9743	-7.57342	-38.4213	-6.27156
24	2VZ6	1	24	-6.26542	3.189135	2 32 13 1	[[45,4,44,47,52,62,1,10,11,20,45,4,44,47,52,62,1,10,11,20],[36,40,42,43,41,39],[29,34,37,70,36,18,26,27,40,71],[80,33,46,61,8,27,71,60,19,28],[42,87,39,40,41,43,86,88,36,84],[55,59,56,58,60,6,14,16,17,68]]	155.5837	-45.4192	-6.82812	-35.3101	-6.26542
24	2VZ6	1	24	-6.16188	2.864801	2 3 6 20	[[29,34,37,47,62,4,10,20,44,45],[10,20,29,44,47,50,62,2,4,7],33,33,[33,26,30,70,75,80,83,48,51,60],[76,31,35,38,75,81,34,36,37,39],[61,73,28,55,66,79,9,14,19,23]]	214.3155	-103.934	-8.94014	-22.495	-6.16188

25	2VZ6	1	25	-6.664	4.577041	1 41 2 1[illegible]	[6, 6, [1, 39, 49, 84, 85, 4, 11, 18, 19, 25, 1, 39, 49, 84, 85, 4, 11, 18, 19, 25], [57, 30, 53, 65, 66, 3, 22, 23, 35, 48], [5, 23, 24, 29, 51, 55, 57, 58, 64, 66], [51, 5, 79, 80, 36, 40, 41, 42, 43, 44], [7, 53, 9, 28, 38, 66, 73, 81, 82, 21]]	377.2727	-71.8013	-8.45308	-30.7546	-6.664
25	2VZ6	1	25	-6.13692	2.438672	2 3 4 5 7[illegible]	[[72, 74, 2, 33, 5, 20, 51, 55, 58, 64], [2, 5, 17, 20, 33, 54, 55, 58, 64, 72], [1, 6, 10, 12, 14, 17, 19, 27, 29, 32], [2, 54, 47, 72, 15, 20, 31, 33, 46, 61], [3, 4, 10, 18, 25, 35, 48, 49, 59, 75], 12, 12, 12, [7, 9, 21, 28, 30, 31, 38, 53, 56, 60], [11, 87, 89, 36, 40, 41, 42, 43, 44, 45]]	547.8917	-81.2818	-9.27258	13.80852	-6.13692
25	2VZ6	1	25	-6.12186	7.345249	2 13 54 5[illegible]	[[10, 69, 86, 32, 34, 39, 41, 43, 49, 87, 10, 69, 86, 32, 34, 39, 41, 43, 49, 87], [3, 35, 44, 58, 75, 76, 88, 90, 5, 23], 5, 5, [5, 51, 64, 29, 8, 23, 24, 57, 58, 62], [79, 8, 37, 52, 80, 6, 27, 29, 64, 78]]	369.5454	-56.4877	-7.1755	-34.9101	-6.12186

25	2VZ6	1	25	-5.95866	4.346958	1 2 3 19	[25,[3,35,76,77,48,22,30,65,66,16],[3,18,25,35,48,49,59,75,76,88],[4,10,11,25,36,40,41,42,49,59],11,11,[43,45,89,91,44,90,41,78,87,40],[79,62,6,27,37,64,80,2,33,72]]	368.196	-107.8	-7.53018	-21.5537	-5.95866
25	2VZ6	1	25	-5.83555	2.668015	1 2 13 5[illegible]	[5,[79,8,37,51,78,5,80,41,43,87],[39,52,84,86,1,4,6,10,11,34],10,10,12,[84,69,85,39,1,10,34,49,12,32],[76,35,75,77,3,48,59,88,90,16]]	354.2467	-48.1088	-7.1863	-29.3469	-5.83555
26	2VZ6	1	26	-6.60549	2.210885	2 3 19 4	[[37,38,40,82,5,8,34,39,41,42],[38,47,82,5,8,10,23,24,29,34],[5,10,22,23,24,34,45,47,55,56],[4,10,15,22,24,33,45,55,57,73],[48,49,1,10,34,2,17,31,67,73],[13,31],[3,43,52,68,16,19,32,44,60,65]]	397.999	-76.9537	-8.7289	-30.7319	-6.60549

26	2VZ6	1	26	-5.89778	3.570346	2	3	17	3[illegible]	[[47,55,76,22,23,56,64,83,8,15],[22,23,45,47,55,56,57,63,76,15],4,4,4,[41,42,85,39,47,83,86,40,37,34],[37,39,41,42,40,38],[2,16],2,31,[2,49,1,6,18,48,50,5,17,26],[77,8,35,37,39,40,41,42,83,85],[31,59,67,68,9,25,30,36,58,65,31,59,67,68,9,25,30,36,58,65]]	449.1767	-113.576	-7.6651	-22.606	-5.89778
26	2VZ6	1	26	-5.8428	3.50126	2	3	13	6	[[56,72,7,22,51,53,55,63,64,70,56,72,7,22,51,53,55,63,64,70],[8,35,47,56,64,76,23,29,77,78],[9,30,36,66,71,79,80,81,3,19],6,6,31,[68,31,43,67,69,11,52,2,13,49],[39,41,42,83,85,34,37,38,40,86],[37,39,41,42,40,38]]	402.1272	-81.4631	-8.0334	-30.7348	-5.8428
26	2VZ6	1	26	-5.77701	3.429343	62	1	41	[illegible]	[31,1,1,[48,69,1,2,49,11,13,16,17,18],[39,48,49,83,37,41,42,85,34,38],[37,39,41,42,40,38],[37,39,41,42,40,38],[4,28,33,45,55,62,63,74,75,15],[47,35,76,77,8,23,29,55,78],7,[76,35,77,78,8,50,34,37,38,39]]	399.4441	-84.1661	-7.67557	-33.4232	-5.77701

26	2VZ6	1	26	-5.58324	6.628027	2	3	40	13	[[47,55,22,23,56,57,78,15,24,29,47,55,22,23,56,57,78,15,24,29],[40,42,55,84,86,41,85,38,39,73],10,[5,6,8,10,49,50,76,35,2,18],[69,2,49,48,1,31,67,68,11,46],[10,34,37,38,40,82,84,39,42,83],[75,68,33,73,4,46,54,74,69,31]]	402.7975	-47.9826	-7.43311	-30.31	-5.58324
27	2VZ6	1	27	-6.89091	3.428299	3	17	18	4	[[5,50,85,87,23,39,41,56,81,35],5,5,[3,5,15,17,22,23,24,38,40,42],1,[4,10,35,51,6,79,38,40,86,49],16,[2,53,61,69,33,73,74,16,18,20]]	256.4904	-42.0939	-7.41796	-33.8246	-6.89091
27	2VZ6	1	27	-6.79982	2.318941	3	4	48	49	[[86,4,5,10,24,35,38,40,50,58],[3,5,15,22,23,24,38,39,41,43],31,11,[49,85,39,41,47,87,35,38,43,77],13,13,[2,44,53,16,20,33,61,74,1,6]]	363.2625	-39.3318	-7.206	-32.2488	-6.79982
27	2VZ6	1	27	-6.66515	2.948341	3	19	33	7	[[33,61,73,74,2,6,20,26,54,63],[6,8,20,22,23,26,28,33,36,54],5,[80,51,8,36,85,79,81,4,10,35],11,11,[10,3,11,31,47,48,68,69,77,1]]	381.5758	-43.7552	-7.62081	-29.1629	-6.66515

27	2VZ6	1	27	-6.22593	3.410486	53 3 19 3	[11,[3,34,46,47,58,76,77,78,4,15],[1,4,10,17,24,35,38,39,41,47],10,[40,42,86,88,43,89,41,10,35,38],[8,28,63,73,26,33,36,51,54,61]]	372.7449	-62.0774	-7.34047	-27.9357	-6.22593
27	2VZ6	1	27	-5.75725	3.068823	2 13 54 5	[[70,71,1,32,47,49,72,40,86,4],[46,56,58,76,78,5,10,22,23,24],55,[5,50,63,81,56,36,79,8,23,28],[64,7,27,29,52,62,65,21,76,56]]	363.4589	-83.7842	-8.42769	-29.6892	-5.75725
28	2VZ6	1	28	-5.98607	4.218321	2 3 29 30	[[34,49,51,2,6,15,16,17,35,36],[2,6,15,34,35,36,39,49,51,1],19,19,[52,59,60,3,8,19,25,38,53,9],[14,40,47,50,52,64,27,46,16,23],[33,72,74,32,73,71,28,31,69,21]]	161.7632	-59.6401	-7.60038	-6.4741	-5.98607
28	2VZ6	1	28	-5.80021	2.145646	2 3 5 7 8	[[13,5,11,39,41,45,65,38,42,43],[5,13,38,39,41,45,54,65,1,3],[20,54,64,45,53,9,13,14,27,46],[36,10,17,21,24,26,34,35,55,57],[12,34,44,49,51,6,15,16,23,43],[16,23,30,50,51,66,67,68,6,14]]	177.247	-53.1539	-7.00441	1.527965	-5.80021

28	2VZ6	1	28	-5.57607	1.955488	4 7 11	[[8,18,19,25,26,52,59,60,29,32],[74,33,72,41,65,29,32,73,53,54],[35,42,55,4,10,11,21,22,36,37]]	140.9987	-44.6685	-7.76043	-26.9894	-5.57607
28	2VZ6	1	28	-5.46307	2.412669	2 3 4 5 6	[[13,39,45,5,12,41,43,1,11,38],[3,5,9,13,38,39,41,45,54,65],[3,8,9,18,19,20,25,26,27,29],[20,54,53,9,13,14,27,45,46,47],21,[36,10,17,24,26,34,35,55,57,61],[49,15,16,44,51,23,34,6,12,43],[16,23,30,50,51,66,67,68,58,24]]	179.9754	-68.5815	-6.77908	1.166421	-5.46307
28	2VZ6	1	28	-5.24232	4.281159	2 13 14 1	[[45,62,13,27,43,63,59,20,38],[4,34,35,36,39,42,44,55,1,2],[24,50,57,58,17,56,51,16,6,23],[31,69,71,28,70,55,10,21,36,26]]	141.0039	-46.2893	-7.36913	-24.6142	-5.24232
29	2VZ6	1	29	-7.06983	2.278996	2 3 17 4	[[56,2,22,37,40,42,47,65,75,3],[2,22,37,39,40,42,44,47,49,55],39,[4,20,24,31,33,36,39,45,49,61],[50,25,34,41,48,77,13,23,43,52]]	434.0211	-94.4771	-9.22329	-22.2562	-7.06983
29	2VZ6	1	29	-6.75824	3.212933	33 34 4 4	[32,32,[1,14,23,32,34,48,52,53,54,70],36,36,[4,39,62,2,37,47,55,56,15,21]]	369.0924	-42.6657	-9.38669	-33.8402	-6.75824

29	2VZ6	1	29	-6.50016	5.764889	2 17 19 7	[[8,10,69,71,6,16],34,[25,27,34,41,44,50,10,16,19,29],[68,7,9,70,6,67,51,83,5,26],[45,49,20,21,39,46,52,24,31,33]]	222.1527	-85.8332	-8.96776	-33.552	-6.50016
29	2VZ6	1	29	-6.44383	2.240024	2 56 3 5	[[34,70,27,44,48,54,1,23,32,45],34,[27,34,41,44,48,54,1,17,18,19],[9,10,16,27,34,41,70,7,71,6],36,36,[49,84,5,24,26,36,40,65,31,33],[62,63,80,81,2,15,37,55,56,4]]	407.6632	-73.8133	-7.81894	-9.11484	-6.44383
29	2VZ6	1	29	-6.21449	1.698579	2 3 17 1	[[42,72,17,22,47,58,75,3,11,12],[17,22,39,42,44,47,4,19,20,21],39,39,[4,20,24,31,33,36,39,45,49,61],[40,44,49,64,65,42,76,5,66,26],[48,23,25,27,32,34,41,50,54,71],[59,3,13,35,77,83,29,64,76,38],33,33,[2,37,53,55,56,78,1,14,15,52]]	421.3815	-76.3525	-9.13037	-16.6607	-6.21449
30	2VZ6	1	30	-6.35791	3.587004	2 13 54	[[55,1,10,13,20,36,61,2,6,19,55,1,10,13,20,36,61,2,6,19],[38,39,75,76,77,78,79,80,35,12],12,12,[12,41,48,95,5,60,93,94,11,40],3,[94,95,96,11,48,49,93,41,97,5]]	199.5613	-38.5235	-5.77957	-35.3912	-6.35791

30 2VZ6	1	30	-6.03488	5.467257	28 2 3 3[illegible]	[[73,9,37,46,74,87,88,2,34,68],[37,73,88,2,9,46,87,89,55,72],[2,37,55,61,72,73,98,6,23,29],13,[12,41,52,54,60,5,21,28,48,93,12,41,52,54,60,5,21,28,48,93]]	114.1714	-64.996	-4.56471	-24.3518	-6.03488
30 2VZ6	1	30	-5.98185	5.480834	2 13 54 [illegible]	[[39,52,60,80,28,54,5,12,18,21],[13,48,93,95,97,10,36,42,45,49],10,10,[10,49,97,98,96,1,36,55],[68,87,63,46,88,9,89,34,51,69]]	223.3679	-56.9637	-3.12104	-29.6711	-5.98185
30 2VZ6	1	30	-5.97013	2.188395	2 3 4 5 [illegible]	[[80,3,39,77,79,86,25,54,57,70],[3,25,39,54,57,79,80,5,16,18],[4,11,12,18,21,26,27,28,33,40],[66,3,57,80,39,78,25,31,33,64],[55,2,20,23,51,53,59,61,19,22],[75,76,30,38,62,63,68,71,88,34],[9,34,46,50,68,69,87,88,99,100],10,[82,83,13,7,42,43,96,98,6,10]]	366.6837	-22.9892	-3.62628	12.17205	-5.97013
30 2VZ6	1	30	-5.93565	4.069573	28 2 56 [illegible]	[[72,68,87,9,34,46,88,89,37,74],[68,72,87,2,53,55,63,75,76,9],10,[2,10,20,23,37,53,55,61,68,72],[84,45,86,96,1,36,85,12,49,98],3,[92,95,4,40,47,58,60,94,11,26]]	107.3473	-56.2131	-5.0647	-15.4497	-5.93565

31	2VZ6	1	31	-7.50212	3.573419	1 2 3 4 5	[19,[40,63,19,28,30,35,47,75,89,8],[19,28,35,40,47,49,63,3,8,13],[4,11,21,27,34,37,38,48,49,61],[42,44,89,91,35,39,40,46,85,43],29,[33,26,73,82,90,18,29,36,74,92],[88,43,45,92,39,42,44,46,89,91],[39,43,45,46,44,42],[39,43,45,46,44,42],[23,25,69,70,71,72,51,56,2,6]]	287.501	-71.5074	-5.49739	-27.4287	-7.50212
31	2VZ6	1	31	-7.30869	2.484534	2 12 13 1	[[49,8,13,47,48,84,1,3,4,18,49,8,13,47,48,84,1,3,4,18],[38,92,34,37,43,45,73,82,83,85],[33,34,38,50,82,37,85,7,10,21],[72,25,70,53,59,6,14,23,58,69],[87,88,33,41,82,86,36,73,71,17],24,[60,15,23,24,32,79,81,6,12,16]]	242.4029	-48.0259	-5.24844	-40.3385	-7.30869
31	2VZ6	1	31	-6.45331	2.191996	3 17 18 1	[[24,32,61,62,79,80,13,16,57,67],13,13,[5,13,16,22,24,48,61,62,66,67],[1,3,4,5,8,13,22,47,48,49],23,23,[56,69,12,23,57,66,5,51,52,54],33,[25,53,71,72,59,7,14,17,50,65]]	261.6761	-50.9598	-5.18071	-36.7257	-6.45331

31	2VZ6	1	31	-6.42199	3.031888	2	12	13	2	[[38,48,49,66,67,8,22,34,47,68,38,48,49,66,67,8,22,34,47,68],[85,83,92,37,43,45,73,82,90,33],[27,33,50,82,85,37,83,7,10,18],23,23,[53,59,69,72,23,25,70,71,14,58],33,[87,88,26,29,33,36,41,73,74,82],[60,16,61,62,80,15,24,32,58,79]]	261.9915	-46.4136	-5.562	-39.3324	-6.42199
31	2VZ6	1	31	-6.40358	3.389037	2	3	13	14	[[82,33,43,73,90,92,26,27,36,39,82,33,43,73,90,92,26,27,36,39],[43,45,87,90,92,35,39,42,44,46],[22,34,37,38,48,49,66,67,68,84],[67,13,52,68,22,5,12,47,54,57],[45,46,92,93,38,43,83,90,39,42],[39,43,45,46,44,42],11,11,[64,81,11,21,62,65,66,24,31,32]]	253.8176	-55.7022	-7.01011	-35.1646	-6.40358
32	2VZ6	1	32	-6.87443	3.713197	2	56	3	19	[[71,2,9,42,50,51,52,73,82,87],42,[9,42,51,52,71,73,87,92,93,2],[11,20,21,22,26,27,33,40,76,86],40,40,[35,43,16,62,84,85,77,11,21,6],[36,64,17,94,95,3,6,28,29,39]]	262.581	-19.6335	-2.55163	-32.1638	-6.87443

32	2VZ6	1	32	-6.53386	3.588915	3	19	4	7	[[51,87,2,16,21,43,50,76,93,11],[2,11,21,27,42,51,76,87,92,93],[7,11,22,40,51,52,66,67,76,77],[16,35,2,50,52,62,66,77,84,11],[6,63,74,10,34,39,75,94,15,17]]	263.7365	-13.1916	-3.93932	-28.7191	-6.53386
32	2VZ6	1	32	-6.14128	2.481603	2	12	13	1	[[75,9,10,42,72,82,91,14,25,26],[2,16,35,43,50,51,87,21,71,76],[7,11,21,66,67,71,76,87,93,73],[37,65,67,78,7,12,18,79,66,61],40,[70,8,68,69,89,5,59,60,61,13]]	269.0805	-26.2085	-6.23271	-27.8102	-6.14128
32	2VZ6	1	32	-6.06606	2.476185	2	13	14	1	[[65,61,71,86,92,7,12,20,66,67,65,61,71,86,92,7,12,20,66,67],[13,25,32,46,59,74,75,80,81,91],[49,1,10,47,75,83,48,74,82,14],[69,70,8,68,74,81,95,36,13,41],35,[50,52,2,35,16,51,43,72,82,83]]	260.2807	4.76986	-2.89784	-34.0512	-6.06606
32	2VZ6	1	32	-5.93977	2.293616	19	11			[[7,11,40,65,66,67,76,77,87,21],[94,95,10,17,28,29,44,47,74,81,94,95,10,17,28,29,44,47,74,81]]	256.6953	-12.2468	-2.79842	-23.4317	-5.93977

33	2VZ6	1	33	-6.29735	1.120934	2 3 4 5 7	[[34,37,38,13,16,18,39,40,59,17],[18,19,34,37,39,40,67,7,11,21],[2,4,11,30,32,44,45,46,47,51],[40,32,37,9,16,17,19,22,27,62],[57,65,5,6,10,36,42,54,64,7],[61,8,33,60,12,28,35,36,41,68]]	268.4001	-101.811	-7.67823	-10.6881	-6.29735
33	2VZ6	1	33	-5.32383	2.154276	2 3 5 7 8	[[34,37,38,39,68,13,16,18,19,26],[18,19,32,34,37,39,40,66,67,11],[32,40,37,16,19,22,24,27,28,69],[6,10,55,57,58,65,7,25,56,1],[35,61,8,33,60,14,36,41,44,63]]	190.7498	-88.1295	-7.45693	9.977112	-5.32383
33	2VZ6	1	33	-5.31965	1.33931	2 12 13 1	[[71,29,53,55,4,5,24,32,52,54],[2,11,45,47,66,67,30,32,46,39],[18,19,34,37,39,40,51,67,4,13],[17,38,33,60,34,27,8,12,16,37],26,26,[7,26,39,59,67,6,56,58,11,18],[49,3,31,48,50,69,27,62,33,12]]	129.5802	-60.4676	-7.60408	-27.5183	-5.31965
33	2VZ6	1	33	-5.13107	1.897782	2 13 14 1	[[16,34,37,38,39,40,13,17,18,19],[9,28,29,51,62,63,70,4,22,24],[43,1,42,44,54,5,71,10,64,65],[3,31,49,50,9,62,63,14,35,44],[66,58,65,11,47,67,2,7,10,21]]	133.0236	-66.0314	-7.84805	-24.9593	-5.13107

33	2VZ6	1	33	-5.10051	1.555056	2	3	4	6	2[illegible]	[[35,49,50,3,9,14,31,62,63,12],[14,35,50,61,62,8,9,12,20,36],[6,7,8,15,23,26,36,56,57,59],28,28,[37,16,17,27,34,38,13,28,40,69],28]	149.248	-54.4718	-7.21833	-13.7488	-5.10051
